# Cost-utility of computed tomography in patients with atypical chest pain clinically referred for invasive coronary angiography: randomised controlled trial

**DOI:** 10.1007/s00330-025-11692-0

**Published:** 2025-05-24

**Authors:** Maria Bosserdt, Mahmoud Mohamed, Konrad Neumann, Nina Rieckmann, Henryk Dreger, Valentin Brodszky, Stefan Höfer, Thomas Reinhold, Anna-Maria Mielke, Marc Dewey

**Affiliations:** 1 Department of Radiology, Charité – Universitätsmedizin Berlin, Freie Universität Berlin and Humboldt-Universität zu Berlin, Berlin, Germany; 2 Institute of Biometry and Clinical Epidemiology, Charité – Universitätsmedizin Berlin, Freie Universität Berlin and Humboldt-Universität zu Berlin, Berlin, Germany; 3 Institute of Public Health, Charité – Universitätsmedizin Berlin, Freie Universität Berlin and Humboldt-Universität zu Berlin, Berlin, Germany; 4 Deutsches Herzzentrum der Charité (DHZC), Berlin, Germany; 5 Department of Health Policy, Corvinus University of Budapest, Budapest, Hungary; 6 Department of Psychiatry II, Medical University Innsbruck, Innsbruck, Austria; 7 Institute of Social Medicine, Epidemiology and Health Economics, Charité - Universitätsmedizin Berlin, Freie Universität Berlin and Humboldt-Universität zu Berlin, Berlin, Germany; 8 Centre for Musculoskeletal Surgery, Charité - Universitätsmedizin Berlin, Corporate Member of Freie Universität Berlin, Humboldt-Universität zu Berlin, Berlin Institute of Health, Berlin, Germany; 9 DZHK (German Centre for Cardiovascular Research), partner site Berlin, Berlin Institute of Health, Berlin, Germany; 10 Berlin University Alliance, Berlin, Germany

**Keywords:** Cost-utility analysis, Coronary artery disease, Computed tomography, Coronary angiography, Quality-adjusted life years

## Abstract

**Background:**

Computed tomography (CT) is as safe as invasive coronary angiography (ICA) in the incidence of major adverse cardiovascular events in patients with atypical chest pain. However, the cost-utility of CT and ICA in healthcare after long-term follow-up is still unknown.

**Methods:**

A prespecified cost-utility analysis (CUA) of 329 patients with atypical chest pain from a single-centre randomised trial compared CT and ICA. The CUA was conducted from the health sector perspective up to a 3-year follow-up using quality-adjusted life years (QALYs) from the EQ-5D-3L questionnaire. Costs were obtained from each individual’s outpatient and inpatient billing data and included cardiovascular medications, hospitalisations, emergency visits, cardiologist visits, and cardiac examinations. The analysis implemented 500 multiple imputations and 1000 bootstrapping iterations per imputed dataset, followed by calculating the net monetary benefit (NMB).

**Results:**

There was no significant difference in mean QALYs at either 1-year (CT: 0.69 (95% CI: 0.66–0.72); ICA: 0.71 (95% CI: 0.68–0.74); difference: −0.02 (−0.06 to 0.03)) or 3-year follow-up (CT: 2.09 (95% CI: 2.00–2.17); ICA: 2.11 (95% CI: 2.02–2.19); difference: −0.02 (95% CI: −0.14 to 0.12)), while the mean cost per patient was significantly lower in the CT compared with the ICA at both 1-year (difference (€): −1647.8, 95% CI: −2198.3 to 1093.3) and at 3-year follow-ups (difference (€): −1543.3, 95% CI: −2228.0 to −830.0). At a willingness-to-pay of €20,000/QALY, the mean incremental NMB of CT over ICA was €1256.5 (164.8–2331.8) at 1-year and €1202.0 (95% CI: −1378.7 to −3961) at 3-year follow-ups.

**Conclusion:**

A CT-first strategy for the management of patients with atypical angina or chest pain was more cost-effective than a direct ICA strategy.

**Trial registration:**

ClinicalTrials.gov NCT00844220.

**Key Points:**

***Question*** *What is the cost-effectiveness of using CT compared to invasive coronary angiography (ICA) for diagnosing coronary artery disease in patients with atypical chest pain?*

***Findings*** *A CT-first diagnostic strategy was €1543 less costly per patient over a 3-year follow-up, yielding similar quality-adjusted life years compared to ICA.*

***Clinical relevance*** *CT offers a cost-effective, non-invasive alternative to ICA for patients with atypical chest pain, reducing healthcare costs significantly without compromising patient-reported outcomes or quality of life.*

**Graphical Abstract:**

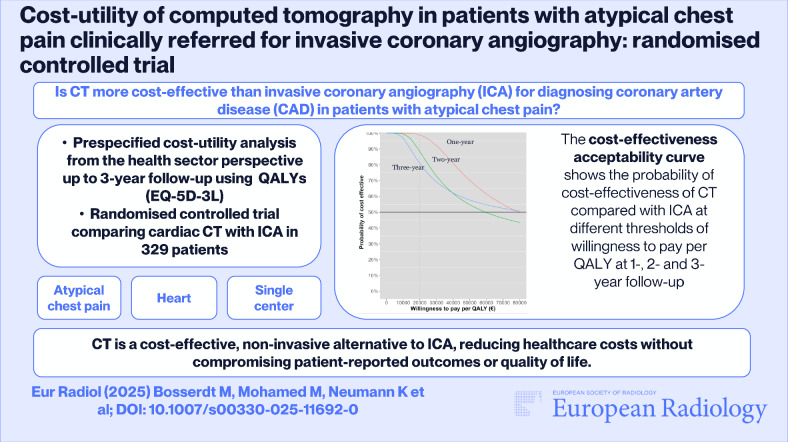

## Background

Coronary computed tomography (CT) angiography is a highly accurate diagnostic test to rule out obstructive coronary artery disease (CAD) in patients with an intermediate pretest probability of CAD [1, 2]. In the SCOT-HEART trial, the addition of CT to standard care halved the incidence of myocardial infarction at 4.8 years of follow-up [3]. Randomised comparisons of CT with invasive coronary angiography (ICA) in the CONSERVE [4] trial at 1-year of follow-up and the CAD-Man [5] and the DISCHARGE [6, 7] trials at 3.3 years and 3.5 years of follow-up, respectively, showed CT to be a safe gatekeeper to ICA that increases the diagnostic yield of ICA with similar major adverse cardiovascular event (MACE) rates.

Simulation studies suggest that in patients with an intermediate pretest probability, CT may be the most cost-effective diagnostic test compared with treadmill ECG, combined dobutamine stress echocardiography and magnetic resonance imaging (MRI), and ICA [8, 9]. Furthermore, microsimulation and cost-minimisation models in patients with acute chest pain presenting to the emergency department show that CT is cheaper than the standard of care [10, 11].

Further cost-effectiveness studies have shown that CT may be more cost-effective than cardiac functional stress tests such as SPECT [12]. Cost analysis of data from the PROMISE trial showed similar costs for CT and functional cardiovascular testing at 3-year follow-up [13], while results of the CONSERVE trial showed lower diagnostic cost of CT compared with ICA at 1-year follow-up [4]. No trial has yet assessed the cost-effectiveness or cost-utility of CT versus ICA.

The aim of this prespecified analysis of the CAD-Man trial was to assess the cost-utility and long-term clinical outcomes of CT versus ICA in patients with atypical chest pain clinically referred for ICA until 3-year follow-up. In addition to the cost-utility analysis (CUA), we analysed patient-reported outcome measures (PROMs), including EuroQoL, Hospital Anxiety and Depression Scale (HADS), and heart disease health-related quality of life (MacNew) for a comprehensive assessment of diagnostic CT versus ICA from the patient’s perspective.

## Methods

### Study design and location

This is a prespecified cost-utility analysis (CUA) of the randomised pragmatic CAD-Man (Coronary Artery Disease Management with Multislice Computed Tomography and Magnetic Resonance Imaging in Patients with Atypical Angina Pectoris) trial (NCT00844220). The trial was conducted at the Charité - Universitätsmedizin Berlin, and the protocol was approved by the institutional ethics committee and by the German Federal Office for Radiation Protection. All participants gave written informed consent before the randomisation. The main results on major procedural complications within 48 h and MACE of the trial and the study protocol have been published previously [5]. The trial compared coronary CT with ICA in patients with atypical chest pain and a clinical indication for ICA because of suspected CAD. The CUA was prespecified on page 18, item 6 of the study protocol. Atypical chest pain was an inclusion criterion for the trial and was defined based on the presence of no more than two out of the three characteristics of typical angina pectoris, as described by Diamond [14]. These characteristics include retrosternal chest discomfort, onset triggered by exertion, and relief within 30 s to 10 min with rest or nitroglycerin. Consequently, patients with atypical angina (meeting two of the three criteria), non-anginal chest pain (meeting one of the three criteria), or other forms of chest discomfort (meeting none of the criteria) were eligible for inclusion in the study [5].

### Participants

The CUA included a total of 329 patients. Patients were included if they had suspected CAD based on atypical angina pectoris and a clinical indication for ICA. Exclusion criteria were: two positive test results for ischaemia, non-sinus rhythm, signs of myocardial infarction (such as persistent ST segment elevation, creatine phosphokinase-MB level > 24 U/L, or pulmonary oedema due to ischaemia), refusal or inability to give informed consent, inability to hold one’s breath for 5 s, age below 30 years, history of CAD (including percutaneous coronary intervention, stenting, and bypass surgery), pregnancy, and haemodialysis treatment.

### Randomisation

Patients were randomised using a computer-generated list (nQuery 7.0; Statistical Solutions) and sequentially numbered, opaque, and sealed envelopes [15].

### Comparators

In the intervention group, coronary CT was performed as described in the study protocol [5]. In case CT was positive for CAD, patients were assigned to ICA for confirmation and treatment. In the control group, ICA was performed as part of standard clinical management, and subsequent patient management decisions were solely based on the ICA findings. CAD was defined as a diameter of 50% or greater in the left main coronary vessel or 70% or greater in another coronary vessel [5].

Detailed management recommendations for the patients in the CT and ICA groups are summarised in the CAD-Man study protocol [5].

### Perspectives

The analytical perspectives for the CUA include healthcare costs in order to capture the consequences of the CT-first versus direct-ICA strategy for the management of patients with atypical angina or chest pain during follow-up. Healthcare costs included costs for cardiovascular medications, hospitalisations, emergency department visits, cardiologist visits, and cardiac examinations (CT, ICA, cardiac MRI, stress ECG, echocardiography, and myocardial scintigraphy). This CUA was performed in accordance with the CHEERS 2022 statement (see Supplementary Material) [16].

### Time horizon

Patients were enrolled between 18 February 2009 and 27 August 2015. The median follow-up of 3.7 years was completed on 27 August 2018, and the retrieval of healthcare costs from hospital and outpatient records was completed in January 2023.

### Outcome measures

The primary outcome, major procedural complications, and the secondary outcome, MACE, were already reported [5]. For the CUA, we used quality-adjusted life years (QALYs) derived from the EQ-5D-3L questionnaire [17] with follow-up at 3 years as the primary effectiveness outcome, as described in the study protocol on page 18, item 6. The EQ-5D-3L questionnaire was completed by the participants prior to the CT or ICA test and at three follow-up time points (6–12 months, 1–2 years, and 3 years after randomisation). QALYs were calculated using the area under the curve method [18].

Due to the low event rates, the outcomes of major procedural complications and MACE, as described in the study protocol, could not be included in this CUA.

### Resource use and costing

Data on cost and resource utilisation were collected from the time of randomisation until dropout, death, or censoring at 3 years. All healthcare costs are in EUR and were reported for the 2009–2018 price year, depending on the year in which the test was performed or the medication was taken.

Information on healthcare services utilisation was collected at the individual patient level for all study participants until a 3-year follow-up after randomisation. At baseline, information on the values reimbursed to the hospital for patients who were inpatients for their initial examinations was obtained from the billing information recorded at Charité, using the actual individual patient coronary DRG (diagnosis-related group; http://www.g-drg.de/). For follow-up, DRG cost was used and calculated with the calculator of the German DRG Research Group (www.drg-reseach-group.de). Cost for outpatient services was calculated using the German reimbursement catalogue (Einheitlicher Bewertungsmaßstab, EBM; https://www.kbv.de/html/ebm.php) for each year (2009–2018). The average cost of the defined daily dose was obtained from the Arzneiverordnungs-Report (an annual publication providing detailed information on prescriptions in Germany) and adjusted to the respective year for the period of 2009–2018 (www.wido.de). Detailed information on the retrieval of cost data and calculations is provided in Supplementary Tables S1–4.

### Cost-utility analysis

The economic analysis was performed in the “intention-to-treat” population from the healthcare perspective. QALY was compared between the CT and ICA groups until 3 years of follow-up. The discount rate for QALYs and cost was 3% per year. Cost-utility was defined as a positive net monetary benefit. Additionally, a cost-effectiveness acceptability curve (CEAC) was generated, and the probability of being cost-effective was estimated at a willingness-to-pay (WTP) threshold of €20,000 according to the NICE guideline [19], and probabilistic cost and QALY differences were plotted on the cost-effectiveness plane.

To calculate QALYs, a multiple imputation algorithm with *n* = 500 samples was implemented for missing EQ-5D scores [20]. Covariates included in the multiple imputation were HADS anxiety and depression scores, time from baseline to visit, gender, age, and cardiovascular risk factors. These covariates were selected because they are known or strongly hypothesised to influence both quality of life in CAD patients (e.g., anxiety, depression, gender [21]) and the likelihood of missing data (e.g., time from baseline to visit). EQ-5D scores at actual visits were adjusted using linear splines with cut points at scheduled visit times. The QALY at each time point was the area under the linear spline curve. If a patient died during follow-up, the post-deathcurve was set to 0 [22]. QALYs and cost were imputed simultaneously in both randomisation groups.

### PROMs

We additionally compared patient-reported outcomes including EQ-5D-3L, HADS, and MacNew between the CT and ICA group. The questionnaires were completed by the participants prior the CT or ICA test and at three time points of the follow-up period (6–12 months, 1–2 years, and 3 years after randomisation) and were previously published in study protocol [5].

Quality of life was assessed using the EQ-5D-3L questionnaire. We used five-dimensional German value sets for the utility scores calculation [17]. The EQ-5D-3L visual analogue scale (EQ VAS) on which the patients rate their own health on the scale from 0 (worst imaginable health state) to 100 (best imaginable health state), was also reported.

The HADS questionnaire assesses the presence and severity of symptoms of anxiety and depression. It consists of seven questions with scores ranging from zero to three. The total score ranges from zero to 21, with a higher score indicating greater depression or anxiety [23].

The MacNew questionnaire [24] assesses the impact of coronary heart disease and its treatment on daily activities and physical, emotional and social functioning. The questionnaire consists of 27 items, which measure three domains of health-related quality of life: physical, emotional and social functioning. In addition, the global score can be calculated. Scale scores range from 1 to 7, with an established minimum important difference of 0.5 MacNew score points.

### Statistical model

Costs and QALYs per randomisation group were calculated, and the incremental cost and QALY were estimated using the seemingly unrelated regressions method to account for the correlation between costs and QALYs [25]. A discount rate of 3% per year after the first year was applied to both costs and QALYs. Neither the incremental cost nor the incremental QALY was adjusted for cardiovascular risk factors in the initial analysis. Results are presented as expected means and 95% confidence intervals. The 95% confidence intervals were calculated based on bias-corrected and accelerated bootstrapping [26] using 1000 replications for each 500 imputed dataset to assess the uncertainty around the estimates. Although the baseline characteristics (e.g., gender, age, cardiovascular risk factor, cardiovascular medical history, and the number of obstructive CAD) were not statistically significantly different between the CT and ICA groups, except for diabetes mellitus (higher in ICA) and pretest probability of CAD (higher in ICA) [5], additional analysis was performed to adjust for age, gender, angina symptoms, diabetes mellitus, hypertension, hyperlipidaemia, family history of CAD, and smoking status.

**Fig. 1:**
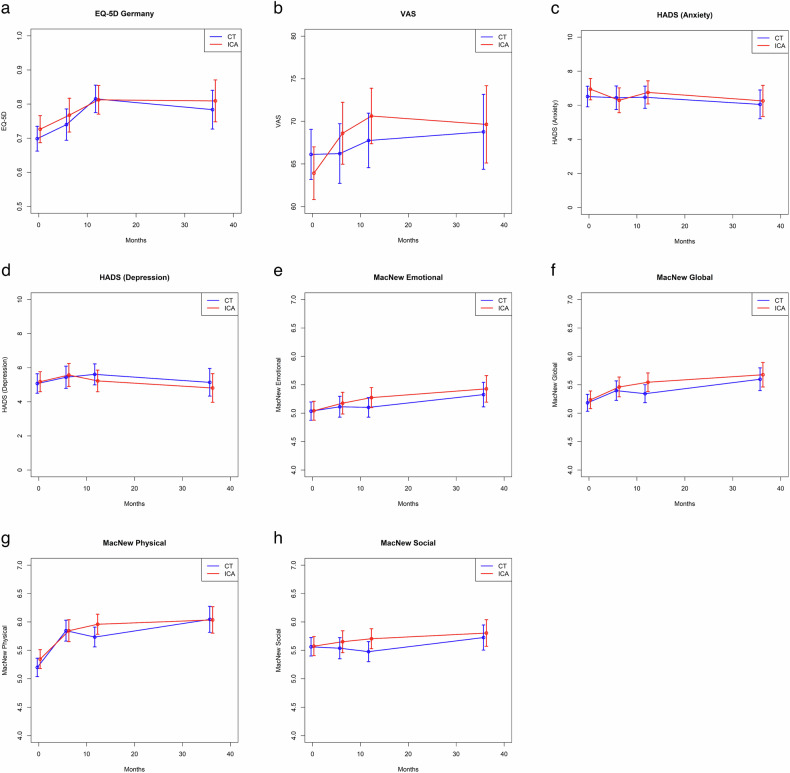
Changes in patient outcomes from baseline to 40-month follow-up in the two groups. Panels show scores over time for EQ-5D-3L Germany (**a**), EQ VAS (**b**), HADS anxiety (**c**), HADS depression (**d**), MacNew emotional (**e**), MacNew global (**f**), MacNew Physical (**g**), and MacNew social (**h**). CT, computed tomography; ICA, invasive coronary angiography; HADS, Hospital Anxiety and Depression Scale; EQ VAS, visual analogue scale. Missing values for each PROM at baseline and three follow-ups are reported in Supplementary Table S5

Means and two-sided 95% confidence intervals for EQ-5D-3L, EQ VAS, HADS anxiety and depression, and MacNew emotional, global, physical, and social scores (presented in Supplementary Table S5 and Fig. 1) were calculated using multiple imputation with *m* = 100 samples. The actual visit times were adjusted to the scheduled visit times at 6–12 months, 1–2 years, and 3 years after randomisation using linear splines.

### Subgroup analyses

Subgroup analyses were performed among clinically important subgroups, including men versus women, patients with and without diabetes mellitus, patients with atypical symptoms versus patients with non-anginal symptoms, and patients with high versus low pretest probability. Pretest probability was calculated using the DISCHARGE calculator [1, 6].

### Sensitivity analysis

A dual approach combining pattern mixture modelling and tipping point analysis was used to assess the robustness of our results to missing data. A different assumption about the missing data mechanism was made for pattern mixture modelling alone: the missing data in the ICA group were assumed to be missing at random (MAR), which contrasted with the assumption that the data in the CT group were missing not at random (MNAR). This distinction was based on an initial assumption that the missing QALY in the CT group was 2% lower than what was observed, denoted as our starting ‘delta’.

The tipping point analysis started with the 2% delta just mentioned and then systematically expanded it in 2% increments. The threshold or ‘tipping point’ was reached when the cost-acceptability curve fell below a 50% probability at a willingness-to-pay (WTP) threshold of €20,000. This approach was applied to 1- and 3-year follow-up data.

Furthermore, probabilistic sensitivity analysis was implemented using nonparametric bootstrapping to represent the statistical uncertainty around the ICERs at 1, 2, and 3 years of follow-up and visualised by plotting the cost-effect pairs on the cost-effectiveness plane. Additional sensitivity analyses were performed, including adjustment for cardiovascular risk factors, since they are known or strongly hypothesised to be associated with both costs and quality of life [21] or were unbalanced between the two groups namely diabetes, application of different discount rates (zero, 1.5%, and 6%), and median-based analyses for both complete cases and imputed data [27].

To further test the robustness of the findings, a pooled imputation approach was applied, incorporating the diagnostic modality (CT vs. ICA) as a covariate in the multiple imputation process to account for potential differences in missingness patterns between the groups. Lastly, a cost-utility analysis (CUA) was conducted, excluding baseline costs and based on the previous pooled imputation approach, to assess the robustness against potential attenuation of cost-effectiveness over time due to anticipated downstream costs beyond the initial management phase.

## Results

### Study population

Supplementary Fig. S1 shows the patient flow and questionnaire completion at baseline and three follow-ups. The CUA included 329 patients (167 in the CT and 162 in the ICA group). Three of the 167 (1.8%) patients in the CT group and one of the 162 patients (0.6%) in the ICA group were lost to follow-up after the procedure, leaving 164 and 160 patients in the CT and ICA groups, respectively (*p* = 0.68). The healthcare-related questionnaire was completed at least once during the 3.7-year follow-up period by 156 of 167 (93%) patients in the CT and by 150 of 162 (92%) patients in the ICA group (Supplementary Fig. S1). The EQ-5D-3L questionnaires were completed at least once during the 3.7-year follow-up by 154 of 167 patients (92%) in the CT and 146 of 162 (90%) patients in the ICA group, while it was completed twice by 149 of 167 (89%) in the CT group and 144 of 162 (89%) in the ICA group (Supplementary Fig. S1).

### Baseline characteristics

Baseline characteristics have been published previously [5]. There were 166 (50.4%) women, and the mean age of the participants was 60.4 years (SD 11.4 years). The overall pretest probability of CAD was 34.3% (SD 23.4%), and CAD was detected in 43 of 329 patients (13%, CT: 18/167 (10.7%), ICA: 25/162 (15.4%, *p* = 0.21)). Thirty-nine of 329 patients (11.8%, CT: 16/167 (9.5%), ICA: 23/162 (14.2%), *p* = 0.2) underwent interventional or surgical revascularisation during initial management [5].

### CUA

The mean QALY difference (CT vs. ICA) was similar at one (−0.02 (95% CI: −0.06 to 0.03)), two (−0.02 (95% CI: −0.11 to 0.06)) and three (−0.02 (95% CI: −0.14 to 0.12)) years of follow-up (Table 1).

**Table 1:** Primary cost-utility analysis of CT versus ICA for baseline management and during the follow-up to 3 years

Analysis	Mean cost (95% CI)* CT group in €	Mean cost (95% CI)* ICA group in €	Difference between CT and ICA in cost (95% CI)* in €	Mean QALYs (95% CI)* in CT group	Mean QALYs (95% CI)* in ICA group	Difference between CT and ICA in QALYs (95% CI)*	Mean NMB of CT at a willingness-to-pay threshold of €20,000 (95% CI)*	Probability of cost-effectiveness at a willingness-to-pay threshold of €20,000
Unadjusted initial management‡ cost	995.8 (799.1–1197.4)	2305.8 (2149.3–2563.4)	−1310.0 (−1632.3 to −1058.7)	n/a	n/a	n/a	n/a	n/a
Unadjusted total cost up to 1 year	1879.8 (1532.3–2292.4)	3527.6 (3120.8–3996.9)	−1647.8 (−2198.3 to −1093.3)	0.69 (0.66–0.72)	0.71 (0.68–0.74)	−0.02 (−0.06 to 0.03)	1256.5 (164.8–2331.8)	99%
Unadjusted total cost up to 2 years	2291.8 (1881.3–2790.2)	3900.2 (3446.5–4421.6)	−1608.4 (−2224.4 to −973.8)	1.4 (1.34–1.46)	1.43 (1.36–1.48)	−0.03 (−0.11 to 0.06)	1135.8 (−649.1 to 2974.9)	89%
Unadjusted total cost up to 3 years§	2582.0 (2133.1–3128.3)	4125.3 (3639.0–4701.9)	−1543.3 (−2228.0 to −830.2)	2.09 (2.00–2.17)	2.11 (2.02–2.19)	−0.02 (−0.14 to 0.12)	1202 (−1378.7 to 3.961)	81%

*CI* confidence interval, *CT* computed tomography, *ICA* invasive coronary angiography, *NMB* incremental net monetary benefit, *ECER* incremental cost-effectiveness ratio, *QALYs* quality-adjusted life years

* The 95% CIs were estimated using bias-corrected and accelerated bootstrapping with 1000 iterations

‡ Initial management-related procedures are reported in the main paper [5]. The DRG coding, DRG text and reimbursed cost at initial management in the two groups are provided in Supplementary Table 1

§ Cost-related procedure and cardiovascular medication consumption at follow-up (until 3 years) are provided in the Supplementary Table 1. The DRG coding, DRG text or EBM numbers and reimbursed costs are provided in Supplementary Tables 2–4

**Fig. 2:**
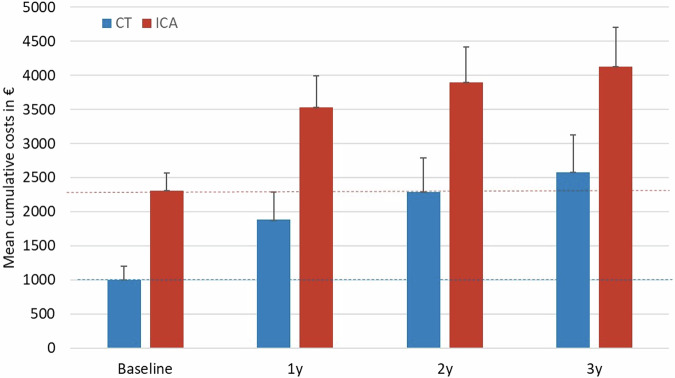
Cumulative total per-patient cost at baseline and up to 1, 2, and 3 years after diagnostic CT or ICA. Included are costs for initial management, inpatient cardiovascular procedures, outpatient procedures and medications. Costs for initial treatment are indicated by dotted horizontal lines (red = ICA; blue = CT)

The unadjusted mean cost difference (CT vs. ICA) decreased little over the follow-up period: it was €−1647.8 (95% CI: €−2198.3 to €−1093.3) at 1 year, €−1608.3 (95% CI: € −2224.4 to €−973.8) at 2 years, and €−1543.3 (95% CI: €−2228.0 to €−830.2) at 3 years follow-up (Table 1 and Fig. 2). The cost-effectiveness plane at 1-, 2- and 3-year follow-ups are shown in Supplementary Fig. S2A–C.

**Fig. 3:**
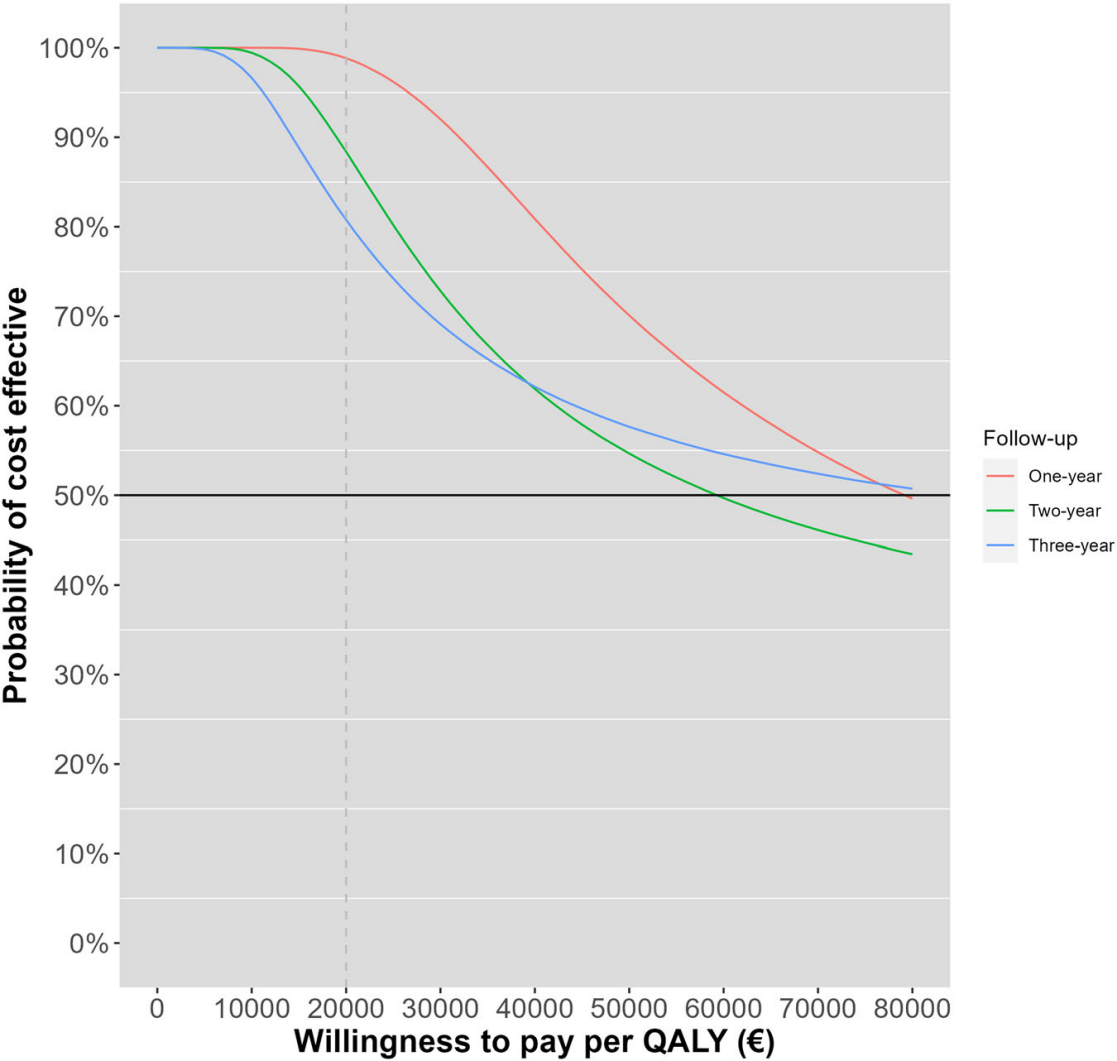
The cost-effectiveness acceptability curve shows the probability of cost-effectiveness of CT compared with ICA at different thresholds of willingness-to pay per QALY at 1-, 2- and 3-year follow-up. QALYs were measured using the EQ-5D-3L (Quality of Life - 5 dimensions) at the appropriate time points. At a willingness-to-pay threshold of €20,000/QALY, CT is cost-effective with 99%, 89% and 81% probability, respectively

The probability of CT being cost-effective at a willingness-to-pay threshold of €20,000/QALY was 99% at 1 year, 89% at 2 years, and 81% at 3 years (Table 1 and Fig. 3), indicating that CT remained cost-effective compared to ICA across all 3 years. The mean incremental net monetary benefit of CT for each year is presented in Table 1.

The unadjusted mean initial management cost was significantly lower in the CT compared with the ICA group (difference: €−1310.2, 95% CI: €−1632.6 to €−1058.7).

The overall cost until 3-year follow-up determined for the three main components was as follows: inpatient cost (CT €650.3 vs. ICA €1064.8), medication cost (CT €896.3 vs. ICA €745.1), and outpatient cost (CT €183.6 vs. ICA €227.9). Cardiovascular disease-related diagnostic and therapeutic procedures after initial management until 3 years are listed in Table 2.

**Table 2:** Comparison of cardiovascular disease-related diagnostic and therapeutic procedures after initial management until 3 years values are absolute numbers (percentages) unless stated otherwise*

	CT group	95% CI	ICA group	95% CI	*p*-value
Inpatient visits during follow-up					
No (%) of cardiovascular-related hospital stays	30 (17.9)	12.1–23.8	33 (20.4)	14.2–26.6	0.58
Total number of cardiovascular-related hospital stays	39		44		
Total number of hospital stays due to chest pain	26		20		
Total number of cardiovascular-related hospital stays due to arrhythmia, tachyarrhythmia and pacemaker implantation/revision	5		19		
Total number of other cardiovascular-related hospital stays	8		5		
No (%) of data missing	8 (4.8)	1.6–8.0	11 (6.8)	2.8–10.3	
Outpatient visits during follow-up					
No (%) of cardiovascular-related emergency department visits	18 (11.5)	6.5 to −16.6	17 (11.3)	6.0–15.8	0.96
Total no (%) of cardiovascular-related emergency department visits	20		17		
No (%) of cardiologist visits	85 (54.9)	46.7–62.3	82 (54.6)	44.7–60.4	0.97
Total no (%) of cardiologist visits	259		316		
No (%) of exercise electrocardiographies	61 (39.1)	31.4–46.8	63 (42.0)	32.7–48.1	0.61
Total number of exercise electrocardiographies	93		128		
No (%) of myocardial scintigraphies	2 (1.3)	−0.5 to 3.0	5 (3.3)	0.4–6.0	
Total number of myocardial scintigraphies	2		6		
No (%) of exercise echocardiographies	17 (10.9)	6.0–15.8	6 (4.0)	0.9–7.5	
Total number of exercise echocardiographies	18		13		
No (%) of computed tomographies	8 (5.1)	1.7–8.6	3 (2.0)	−0.2 to 4.1	
No (%) of cardiac magnetic resonance imaging examinations	4 (2.6)	0.1–5.0	3 (2.0)	−0.2 to 4.1	
No (%) of data missing	11/167 (6.6)	2.8–10.3	12/162 (7.4)	3.4–11.4	
Medication during follow-up‡					
No (%) of lipid-lowering agents:	86 (57.3)	49.4–65.2	66 (46.2)	38.0–54.3	0.062
Mean days	470 ± 468	2.0–938	376 ± 462	−86 to 838	0.085
Antianginal agents					
No (%) of beta-blockers	94 (62.7)	54.8–70.4	85 (59.4)	51.4–67.5	0.632
Mean days	564 ± 501	63–1065	527 ± 501	26.0–1028	0.534
No (%) of calcium-channel blockers	36 (24.0)	17.2–30.8	45 (31.5)	23.9–39.1	0.191
Mean days	205 ± 389	−184 to 594	225 ± 375	−150 to 600	0.664
No (%) of nitrates	6 (4.0)	0.9–7.1	9 (6.3)	2.3–10.3	0.434
Mean days	18 ± 107	−89 to 125	28 ± 123	−95 to 151	0.475
No (%) of other antianginals	10 (6.7)	2.7–10.7	17 (11.9)	6.6–17.2	0.157
Mean days	57 ± 224	−167 to 281	83 ± 250	−167 to 333	0.334
No (%) of antiplatelet agents	71 (47.3)	39.3–55.3	76 (53.1)	45.0–61.3	0.351
Mean days	358 ± 433	−75 to 791	435 ± 469	−34 to 904	0.149
Antihypertensive agents					
No (%) of ACE inhibitors	41 (27.3)	20.2–34.5	53 (37.1)	29.1–45.0	0.081
Mean days	213 ± 382	−169 to 595	273 ± 416	−143 to 689	0.201
No (%) of ARBs	64 (41.6)	34.8–50.6	52 (35.9)	28.5–44.2	0.343
Mean days	326 ± 431	−105 to 757	312 ± 449	−137 to 761	0.771
No (%) diuretics	46 (30.7)	23.3–38.0	42 (29.4)	21.9–36.8	0.899
Mean days	249 ± 409	−200 to 698	228 ± 391	−163 to 619	0.660
No (%) of other antihypertensives	3 (2.0)	−0.2 to 4.2	1 (0.7)	−0.7 to 2.1	0.623
No (%) anticoagulants	22 (14.7)	9.0–20.3	19 (13.3)	7.7–18.8	0.740
Mean days	126 ± 325	−199 to 451	111 ± 306	−195 to 417	0.687
No (%) of data missing	17 (10.2)	6.3–16.4	19 (11.7)	7.7–18.8	

*CT* computed tomography, *ICA* invasive coronary angiography, *CI* confidence interval, *ACE* angiotensin-converting enzyme, *ARB* angiotensin-receptor blocker

* Data on cost-related procedures at initial management are reported in the main paper [5], and results for hospital stays at initial management are provided in Supplementary Table 1

‡ Cardiovascular drug intake at least one day during follow-up

### Subgroup analysis

At a WTP threshold of €20,000/QALY, CT was cost-effective for all subgroups (Table 3). At the €20,000 WTP/QALY threshold, women (79%) and patients with pretest probability of > 30% (80%) were most likely to benefit from CT. In contrast, only 54% of patients with diabetes mellitus benefited from CT (Table 3).

**Table 3:** Subgroups cost-utility analyses of CT versus ICA by subgroup (cost up to 3 years)

Analysis	Mean cost (95% CI)* CT group in €	Mean cost (95% CI)* ICA group in €	Difference between CT and ICA in cost (95% CI)* in €	Mean QALYs (95% CI)* in CT group	Mean QALYs (95% CI)* in ICA group	Difference between CT and ICA in QALYs (95% CI)*	Mean NMB of CT at a willingness-to-pay threshold of €20,000 (95% CI)*	Probability of cost-effectiveness at a willingness-to-pay threshold of €20,000
Women	2446.1 (1946.7–3048.9)	4093.7 (3356.7–5017.0)	−1647.6 (−2705.3 to −691.4)	2.02 (1.91–2.14)	2.04 (1.91–2.15)	−0.01 (−0.18 to 0.15)	911.8 (−2645.8 to 4659.2)	79%
Men	2738.7 (2058.2–3668.2)	4121.8 (3561.1–4894.6)	−1383.1 (−2385.2 to −293.3)	2.16 (2.04–2.27)	2.19 (2.06–2.3)	−0.03 (−0.19 to 0.15)	800.3 (−2706.1 to 4480.2)	69%
Pretest probability of coronary artery disease‡ (≤ 30%)	2307.3 (1720.8–3078.1)	4033.9 (3385.5–4936.6)	−1726.7 (−2822.3 to −743.1)	2.16 (2.01–2.28)	2.2 (2.06–2.33)	−0.05 (−0.23 to 0.15)	896.5 (−3158.2 to 5084.9)	66%
Pretest probability of coronary artery disease‡ (> 30)	2766.5 (2191.9–3581.6)	4169.7 (3515.7–4986.1)	−1403.2 (−2340.8 to −381.8)	2.05 (1.95–2.15)	2.05 (1.94–2.16)	0 (−0.16 to 0.15)	1445.6 (−1803.1 to 4637.0)	81%
Atypical angina	2558.2 (1893.4–3411.2)	4080.4 (3386.4–4982.6)	−1522.2 (−2654.5 to −365.9)	1.97 (1.81–2.11)	2.02 (1.89–2.14)	−0.06 (−0.24 to 0.15)	461.1 (−3666.0 to 4818.6)	58%
Non-anginal chest pain	2597.0 (2065.1–3335.5)	4169.3 (3522.7–4985.5)	−1572.3 (−2528.9 to −613.4)	2.17 (2.07–2.26)	2.2 (2.08–2.3)	−0.03 (−0.17 to 0.12)	1124.6 (−1991.2 to 4207.8)	76%
With diabetes mellitus	2661.8 (1487.2–4489.4)	4729.5 (3606.3–6226.8)	−2067.7 (−3850.9 to 8.9)	1.81 (1.51–2.09)	1.91 (1.68–2.11)	−0.09 (−0.46 to 0.27)	349.2 (−7422.8 to 8099.2)	54%
Without diabetes mellitus	2581.0 (2102.9–3160.8)	3993.1 (3455.5–4626.9)	−1412.0 (−2183.4 to −655.4)	2.12 (2.03–2.21)	2.16 (2.06–2.24)	−0.04 (−0.16 to 0.09)	639.8 (−2002.2 to 3425.5)	67%

*CI* confidence interval, *CT* computed tomography, *ICA* invasive coronary angiography, *NMB* incremental net monetary benefit, *ECER* incremental cost-effectiveness ratio, *QALYs* quality-adjusted life years

* The 95% CIs were estimated using bias-corrected and accelerated bootstrapping with 1000 iterations

‡ The pretest probability was calculated using the CoMe-CCT calculator [1]

### Sensitivity analysis

The tipping point analysis at the 3-year follow-up showed that the missing QALY values in the CT group needed to be at least 14% below their observed values to change our conclusion (Supplementary Fig. S3). Conversely, at 1-year follow-up, the missing QALY values in the CT group should be at least 54% lower than their observed values to have a similar impact on the conclusion (Supplementary Fig. S4).

When comparing analyses that were unadjusted with analyses that were adjusted for age, gender, symptoms, diabetes mellitus, hyperlipidemia, arterial hypertension, smoking, and family history of CAD, we observed that the probability of CT being cost-effective decreased from 99 to 98% at 1-year follow-up and from 89 to 82% at 2-year follow-up, while it decreased from 81 to 70% at 3-year follow-up using the €20,000 WTP threshold per QALY (Table 4). The findings were robust against the further sensitivity analyses, including median-based analysis, median-based complete case analysis, the pooled imputation approach, and the discount rates of zero, 1.5 and 6% showed similar effects as the base case analysis (Table 4). Despite the attenuation in cost difference after the initial management, excluding the baseline cost resulted in the probability of CTA being cost-effective, fluctuating between 48% and 52% at €20,000 WTP (Table 4).

**Table 4:** Sensitivity cost-utility analysis of CT versus ICA for baseline management and during the follow-up to 3 years

Analysis	Mean cost (95% CI)* CT group in €	Mean cost (95% CI)* ICA group in €	Difference between CT and ICA in cost (95% CI)* in €	Mean QALYs (95% CI)* in CT group	Mean QALYs (95% CI)* in ICA group	Difference between CT and ICA in QALYs (95% CI)*	Mean NMB of CT at a willingness-to-pay threshold of €20,000 (95% CI)*	Probability of cost-effectiveness at a willingness-to-pay threshold of €20,000
Adjusted** total cost up to 1 year	2582.2 (2133.1–3128.4)	4124.7 (3638.4–4700.6)	−1642.2 (−2182.9 to −1083.0)	0.69 (0.66–0.72)	0.71 (0.68–0.74)	−0.03 (−0.07 to 0.02)	1117.2 (12.7–2195.1)	98%
Adjusted** total cost up to 2 years	2291.8 (1881.3–2790.2)	3900.2 (3446.5–4421.6)	−1608.4 (−2224.4 to −973.8)	1.4 (1.34–1.45)	1.42 (1.36–1.48)	−0.02 (−0.10 to 0.06)	1135.8 (−649.1 to 2975.0)	82%
Adjusted** total cost up to 3 years	2582.2 (2133.1–3128.4)	4125.8 (3639.3–4702.9)	−1560.1 (−2251.2 to −821.3)	2.09 (2.0–2.18)	2.11 (2.02–2.19)	−0.05 (−0.16 to 0.08)	713.7 (−1876.3 to 3393.5)	70%
Median-based analysis (unadjusted)	1371.3 (1090.1–1588.3)	2819. (2503.8–3129.4)	−1448.0 (−1875.3 to −1008.0)	2.14 (2.05–2.23)	2.17 (2.07–2.22)	−0.04 (−0.148 to 0.09)	801.1 (1583.0–3393.9)	72%
Median-based complete case analysis (unadjusted)	1518.44 (1313.8–3022.8)	2969.9 (2535.6–3226.2)	−1451.4 (−1944.7 to −327.5)	2.3 (2.2–2.4)	2.3 (2.2–2.4)	−0.01 (−0.14 to 0.15)	1258.7 (−1526.3 to 4299.2)	82%
0% discount rate	2732.7 (2257.5–3310.9)	4373.5 (3858.4–4983.7)	−1640.8 (−2365.2 to −886.7)	2.19 (2.10–2.27)	2.21 (2.11–2.30)	−0.021 (−0.146 to 0.11)	1302.6 (−1349.8 to 4133.3)	82%
1.5% discount rate	2656.8 (2194.8–3218.8)	4248.5 (3747.8–4841.6)	−1591.7 (−2296.0 to −858.3)	2.14 (2.05–2.22)	2.16 (2.06–2.25)	−0.02 (−0.14 to 0.11)	1250.4 (−1338.2 to 4014.4)	82%
6% discount rate	2435.5 (2012.0–2950.4)	3884.3 (3425.9–4428.6)	−1448.8 (−2095.0 to −776.0)	2.00 (1.91–2.07)	2.01 (1.93–2.1)	−0.02 (−0.14 to 0.10)	1099.8 (−1308.3 to 3669.4)	80%
Pooled imputation at 1-year follow-up	1884.5 (1536.5–2295.5)	3492.0 (3094.7–3927.9)	−1607.6 (−2169.5 to −1017.4)	0.694 (0.66–0.72)	0.71 (0.68–0.74)	−0.02 (−0.06 to 0.03)	1287.64 (228.9–2349.8)	99%
Pooled imputation at 2-year follow-up	2301.1 (1887–2809.9)	3861.4 (3397.5–4372.5)	−1560.4 (−2214.1 to −858.1)	1.41 (1.35–1.46)	1.42 (1.36–1.48)	−0.02 (−0.09 − 0.07)	1224.9 (−471.1 to 3023.1)	92%
Pooled imputation at 3-year follow-up	2580.7 (2127.5–3141.3)	4115.3 (3631.9–4690.1)	−1534.5 (−2217.4 to −814.2)	2.10 (2.01–2.18)	2.11 (2.02–2.2)	0.01 (−0.13 − 0.11)	1418.0 (−1122.9 to 4061.7)	86%
Baseline cost exclusion at 1-year follow-up	883.1 (636.1–1245.8)	1197.8 (872.3–1569.0)	−314.695 (−775.1 to 142.3)	0.694 (0.66–0.72)	0.71 (0.68–0.74)	−0.02 (−0.06 − 0.03)	−5.329 (−993.2 to 965.6)	50%
Baseline cost exclusion at 2-year follow-up	1329.7 (1002.875–1783.9)	1636.0 (1237.7–2090.5)	−306.273 (−870.5 to 271.0)	1.41 (1.35–1.46)	1.42 (1.36–1.48)	−0.02 (−0.09 − 0.07)	−29.2 (−1665.6 to 1686.1)	48%
Baseline cost exclusion at 3-year follow-up	1647.8 (1265.2–2134.1)	1933.4 (1482.7–2455.6)	285.5 (–924.5 to 362.5)	2.10 (2.01–2.18)	2.11 (2.02–2.2)	0.01 (−0.13 to 0.11)	94.28 (−2260.0 to 2625.8)	52%

*CI* confidence interval, *CT* computed tomography, *ICA* invasive coronary angiography, *NMB* incremental net monetary benefit, *ECER* incremental cost-effectiveness ratio, *QALYs* quality-adjusted life years

* The 95% CIs were estimated using bias-corrected and accelerated bootstrapping with 1000 iterations

** Adjusted for age, gender, symptoms, diabetes mellitus, hyperlipidaemia, arterial hypertension, smoking and family history of CAD

### PROMs

At baseline and during follow-up at all three time points (6–12 months, 1–2 years, and 3 years after randomisation), PROMs such as EQ-5D-3L, HADS and MacNew were similar in the CT and ICA groups (Supplementary Table S5, Fig. 1). Missing values for each PROM at baseline and three follow-ups are reported in Supplementary Table S5.

## Discussion

### Main findings

The paper presents the cost-effectiveness analysis of CT vs. ICA in the single-centre randomised CAD-Man trial until 3 years of follow-up in patients with atypical angina or chest pain referred for invasive coronary angiography. CT was superior to ICA, as we observed a similar level of QALYs in the CT group and ICA group, while the level of cost was significantly lower in the CT group than in the ICA group (difference (CT-ICA): €−1543.3). The mean incremental net monetary benefit for CT vs. ICA was €1256.5 at 1 year and €1202.0 at 3 years. The probability of CT being cost-effective compared with ICA was 80% at a willingness-to-pay threshold of €20,000. CT- and ICA-guided strategies showed similar improvements in PROMs (EQ-5D-3L, HADS and MacNew) from baseline to follow-up at a median of 3.7 years.

Initial management costs were significantly lower for CT than for ICA. This cost difference can be attributed to the lower direct cost of CT, as previously indicated in simulation studies using decision analytical modelling [8, 9, 28]. In Germany, typically, CT is performed in an outpatient setting, whereas ICA requires a hospital stay of at least three days, which also explains the cost differences at initial management. Furthermore, DRG costs are lower when CT is performed during hospitalisation, as CT is less expensive compared to ICA. Although the hospitalisation rates at the follow-up were similar in the two groups, ICA resulted in higher hospitalisation cost as more patients were admitted to the cardiology department for reasons such as arrhythmia, tachyarrhythmia, and pacemaker implantation/revision, which are associated with higher cost than chest pain management.

Additionally, CT resulted in fewer revascularisations (9% vs. 14%) and a lower need for angiography (from 100 to 14%) [5], contributing to a substantial 57% reduction in initial management cost compared with ICA. Our findings remained robust across all sensitivity analyses. Even after excluding these baseline costs, ICA did not surpass CT in terms of cost-effectiveness.

Both CT- and ICA-guided strategies yielded comparable overall numbers of inpatient and outpatient visits, examinations and amounts of medication use during the follow-up period of up to 3 years. Consequently, the cost disparity between CT and ICA remained consistent over the 3-year follow-up period, with differences (CT-ICA) of €1647.8 at 1 year, €1608.3 at 2 years and €1543.3 at 3 years.

When considering only medication cost during follow-up, we found CT to result in a slightly higher cost than ICA. The increased medication cost in the CT group can be attributed to the 11% higher usage of statins in this group than in the ICA group. This is consistent with findings of the SCOT-HEART trial, which showed that CT led to a greater change in treatment with higher statin use compared with standard care [29].

### Comparison with literature

Our findings are in line with the results of the CONSERVE trial [4], which compared CT with a direct referral strategy using ICA as index test for patients with stable chest pain but suspected CAD. In that trial, the investigators found that using CT as the initial test reduced total cost by 57% compared with ICA at 1-year follow-up. However, the investigators only analysed the cost of invasive and non-invasive testing, while they did not include medication or hospitalisation cost at follow-up and did not evaluate EQ-5D-3L for a full CUA. Also, in the CAT-CAD trial [30], the use of CT as the initial diagnostic test in stable patients with an indication for ICA led to a significant cost reduction. The trial demonstrated a 63% decrease in the cost associated with diagnosing CAD and a 55% reduction in the combined cost of diagnosis and treatment during the 90-day follow-up period. We identified no other studies that specifically analysed the cost of CT and ICA in patients with suspected CAD.

Several randomised trials have compared the cost of CT with different comparators, such as standard care [10, 11, 31–33], functional testing [13, 34, 35], or myocardial perfusion single-photon emission CT [36] in patients with stable chest pain or suspected CAD. In almost all the trials, CT was less expensive [10–12, 30, 32, 34–36] or had the same cost as the comparator [13, 34]. Only in the RAPID-CTCA trial [31], which compared the use of early CT in addition to standard care with standard care alone in patients with suspected or provisionally diagnosed acute coronary syndrome, were the costs of CT higher than those of standard care alone. The investigators attributed the higher cost in the CT group to the inclusion of patients from the emergency department and the additional use of CT, which resulted in longer hospital stays and additional CT scans. More detailed information on the trials can be found in Supplementary Table S6.

### Limitations

As this trial enrolled patients with suspected CAD presenting with atypical angina or chest pain, relatively low rates of obstructive CAD were detected by CT and ICA. While this low proportion of obstructive CAD in our study population may have resulted in reported cost that are lower than actual healthcare cost in general patient populations, the comparable prevalence of obstructive CAD in the CT and ICA groups allows a meaningful cost comparison in the patient population investigated.

This analysis considered only the cost from the health sector perspective, potentially overlooking other important costs such as travel expenses or societal costs. The exclusion of such costs could impact the overall cost comparison between CT and ICA, although the lower rate of initial hospitalisations in the CT group and no increase in hospitalisations during follow-up make this unlikely.

It must be acknowledged that our limited sample size contributed to the wide confidence intervals around the NMB, particularly evident at 3-year follow-up and in the subgroup analyses. Despite these observations, the tipping point analysis revealed that an exceedingly extreme scenario would have to be met for ICA to surpass CT in terms of cost-effectiveness. Notably, the tipping point analysis at 1-year follow-up revealed that for ICA to be more cost-effective, the missing QALY values in the CT group would need to be at least 54% lower than actually observed to make ICA more cost-effective.

The proportion of patients with diabetes mellitus was low (9% vs. 18.5%) and significantly different between the CT group and the ICA group. This imbalance might have confounded our cost comparison. However, it is worth noting that, in the subgroup of patients with diabetes mellitus, the probability of CT being cost-effective compared with ICA was 52% at a willingness-to-pay threshold of €20,000. This suggests that, even with the imbalance in diabetes mellitus prevalence, CT may remain a favourable, cost-effective option for this subgroup of patients.

A direct-ICA strategy for patients with suspected CAD presenting with atypical angina does not reflect the standard diagnostic approach in most countries, where non-invasive testing is typically performed first. This limits the generalisability of our findings to healthcare settings where ICA is used as an initial diagnostic test. Further cost analyses should be performed in multicentre studies in different countries to overcome the generalisability issue.

It is important to note that this analysis is based on a single-centre trial conducted at Charité. The clinical settings and cost may differ in other centres, both in Germany and in other countries. However, the findings of the CONSERVE trial [4] conducted in Korea support our results, demonstrating lower cost for CT than for ICA.

## Conclusion

A CT-first strategy for managing patients with atypical angina or chest pain is a cost-effective, non-invasive alternative to ICA. Overall, a 3-year follow-up, CT reduced overall healthcare costs by €1543 per patient while maintaining similar QALY and patient-reported outcomes. At a willingness-to-pay threshold of €20,000, the probability of CT being cost-effective compared to ICA was 81%. These findings further support current guideline recommendations favouring CT as a first-line diagnostic strategy in this patient population.

## Supplementary information

ELECTRONIC SUPPLEMENTARY MATERIAL

## Abbreviations

CAD: Coronary artery disease
CT: Computed tomography
CUA: Cost-utility analysis
DRG: Diagnosis-related group
EQ VAS: EuroQol visual analogue scale
HADS: Hospital Anxiety and Depression Scale
ICA: Invasive coronary angiography
MACE: Major adverse cardiovascular event
MacNew: MacNew Heart Disease Health-related Quality of Life questionnaire
MRI: Magnetic resonance imaging
PROMs: Patient-reported outcome measures
QALY: Quality-adjusted life year
SD: Standard deviation
WTP: Willingness-to-pay
