# Supplementary material for: Cost-utility of computed tomography in patients with atypical chest pain clinically referred for invasive coronary angiography: randomised controlled trial
Source: Eur Radiol. 2025 May 24;35(11):6719–34. doi: 10.1007/s00330-025-11692-0 (PMC12559085; doi:10.1007/s00330-025-11692-0)
Supplement: Supplementary file 1 — ELECTRONIC SUPPLEMENTARY MATERIAL [file 330_2025_11692_MOESM1_ESM.pdf]

# Cost-utility of computed tomography in patients with atypical chest pain clinically referred for invasive coronary angiography: randomised controlled trial

## ELECTRONIC SUPPLEMENTARY MATERIAL

**Appendix 1: Additional data and results**


**Fig S1 Flowchart of patients in study including follow-up for healthcare and quality of life endpoints**

**Fig S2A Cost-effectiveness plane at one-year follow-up**

**Fig S2B Cost-effectiveness plane at two-year follow-up**

**Fig S2C Cost-effectiveness plane at three-year follow-up**

**Fig S3 Dual approach combining pattern mixture modelling with tipping point analysis at three-year follow-up**

**Fig S4 Dual approach combining pattern mixture modelling with tipping point analysis at one-year follow-up**

**Table S1 Reported DRG codings, DRG texts and reimbursed costs at baseline in the two groups**

**Table S2 Reported DRG codings, DRG texts and reimbursed costs for cardiovascular-related hospitalisations at follow-up (until 3 years) in the two groups**

**Table S3 EBM numbers and costs of outpatient healthcare services**

**Table S4 Average cost in € per DDD (defined daily dose) for each year**

**Table S5 Comparison of patient-reported outcomes at baseline and at the three follow-up time points (median follow-up of 3.7 years).**

**Table S6 Comparison of randomised trials (including at least one group undergoing CT) investigating costs in patients with stable chest pain or suspected coronary artery disease**

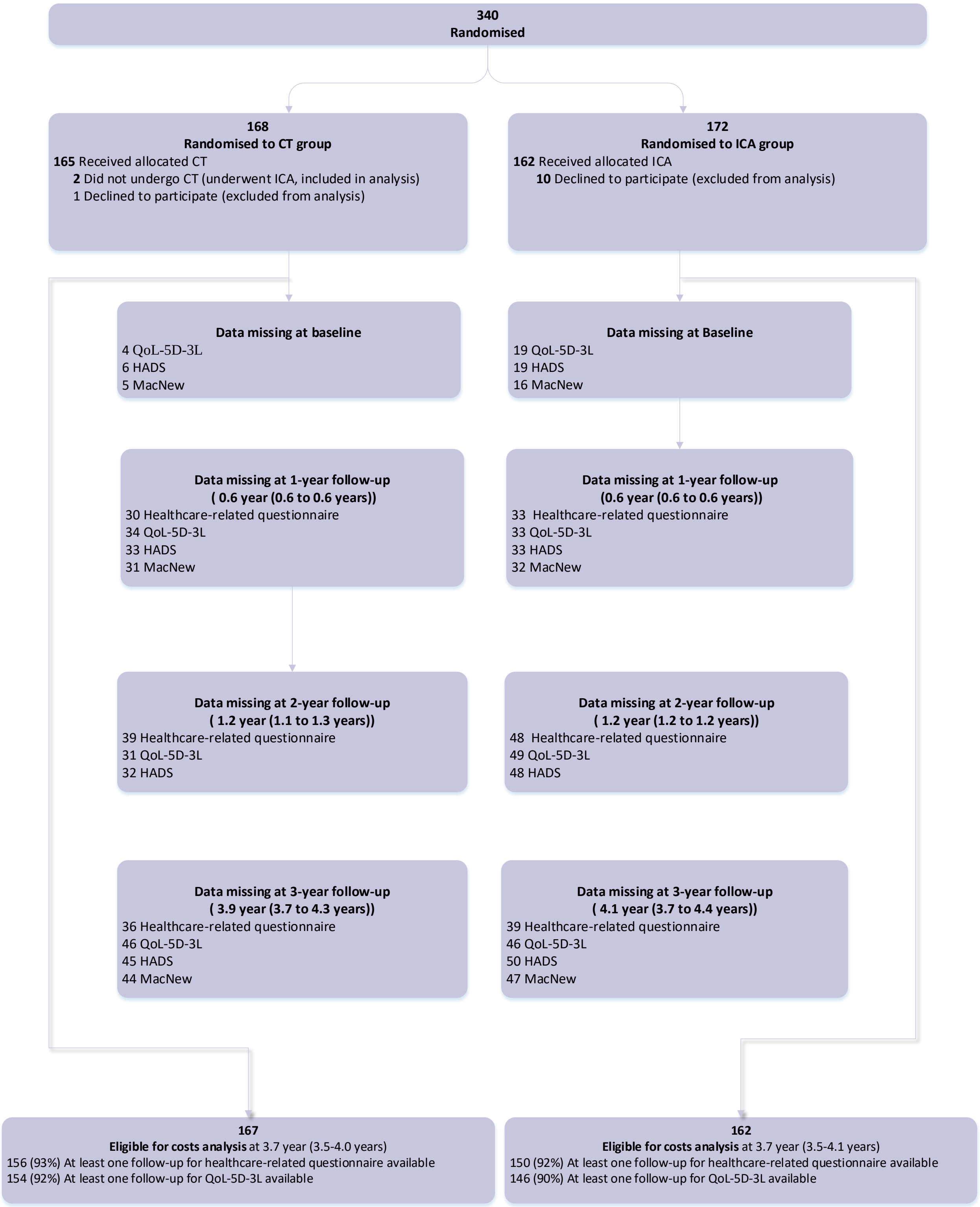


**Fig S1** Flowchart of patients in study including follow-up for healthcare and quality of life endpoints

The EQ-5D-3L questionnaires were completed at least once during the 3.7-year follow-up by 154 of 167 patients (92%) in the CT group and 146 of 162 patients (90%) in the ICA group, while it was completed twice by 149 of 167 (89%) in the CT group and 144 of 162 (89%) in the ICA group.

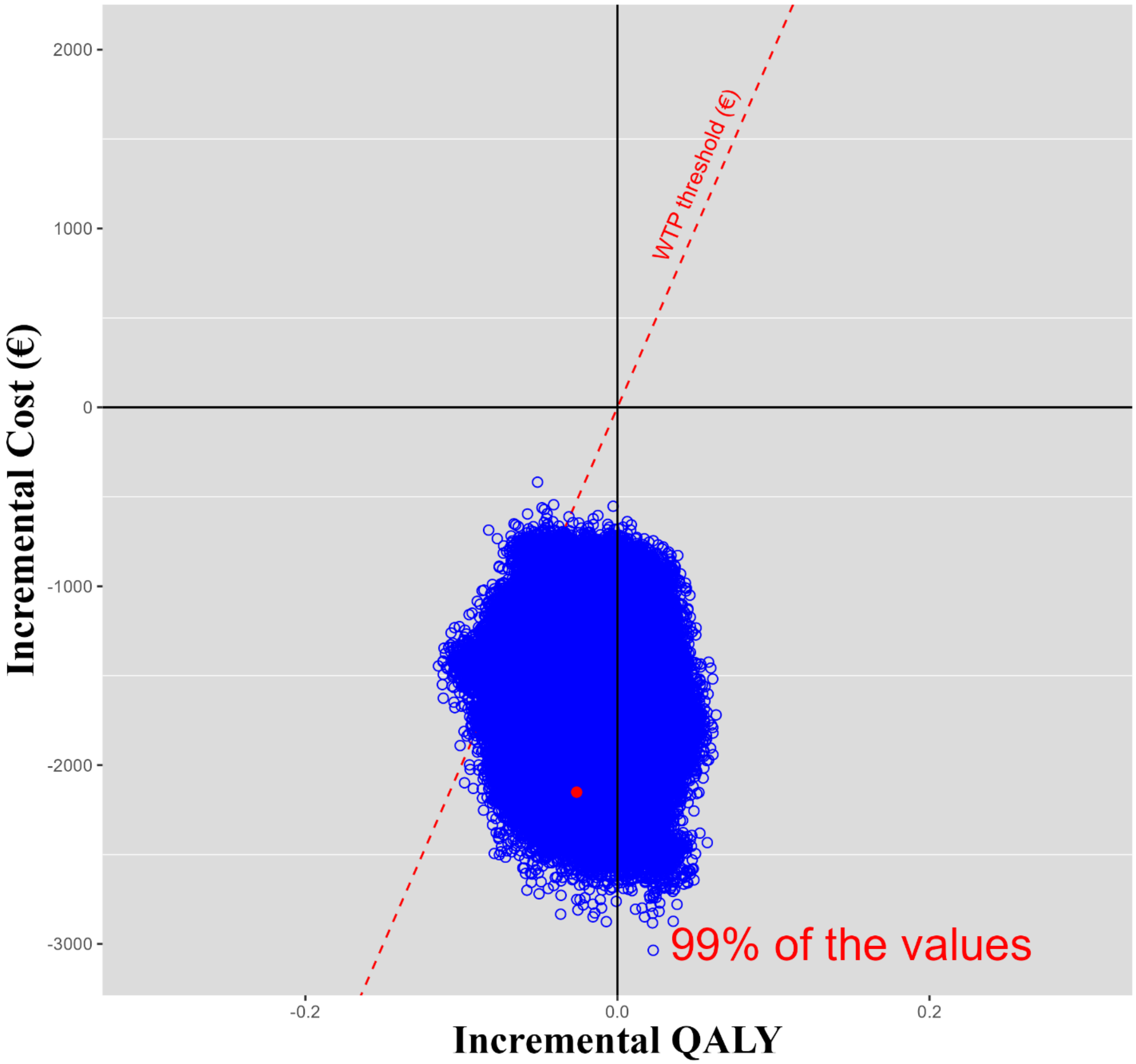


**Fig S2A** Cost-effectiveness plane at the one-year follow-up

Probabilistic sensitivity analysis of cost-effectiveness at one-year follow-up performed using 1000 bootstraps for each imputed dataset (500,000 circles in total). Blue circles depict the uncertainty surrounding the ICER (red) point.

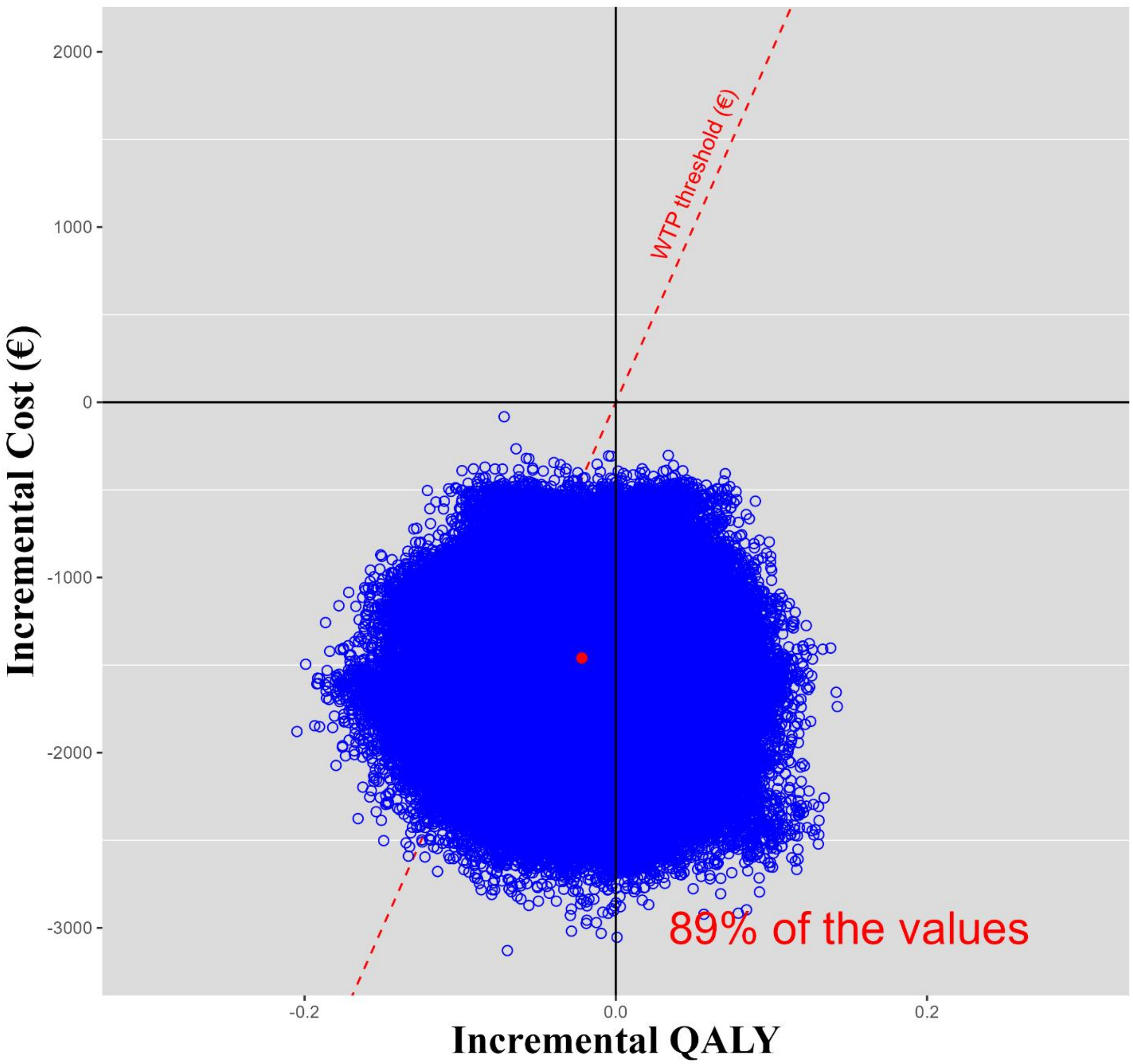


**Fig S2B** Cost-effectiveness plane at two-year follow-up

Probabilistic sensitivity analyses of cost-effectiveness at the two-year follow-up performed by 1000 bootstraps for each imputed dataset (500,000 circles in total), which depicts the uncertainty surrounding the ICER (red) point.

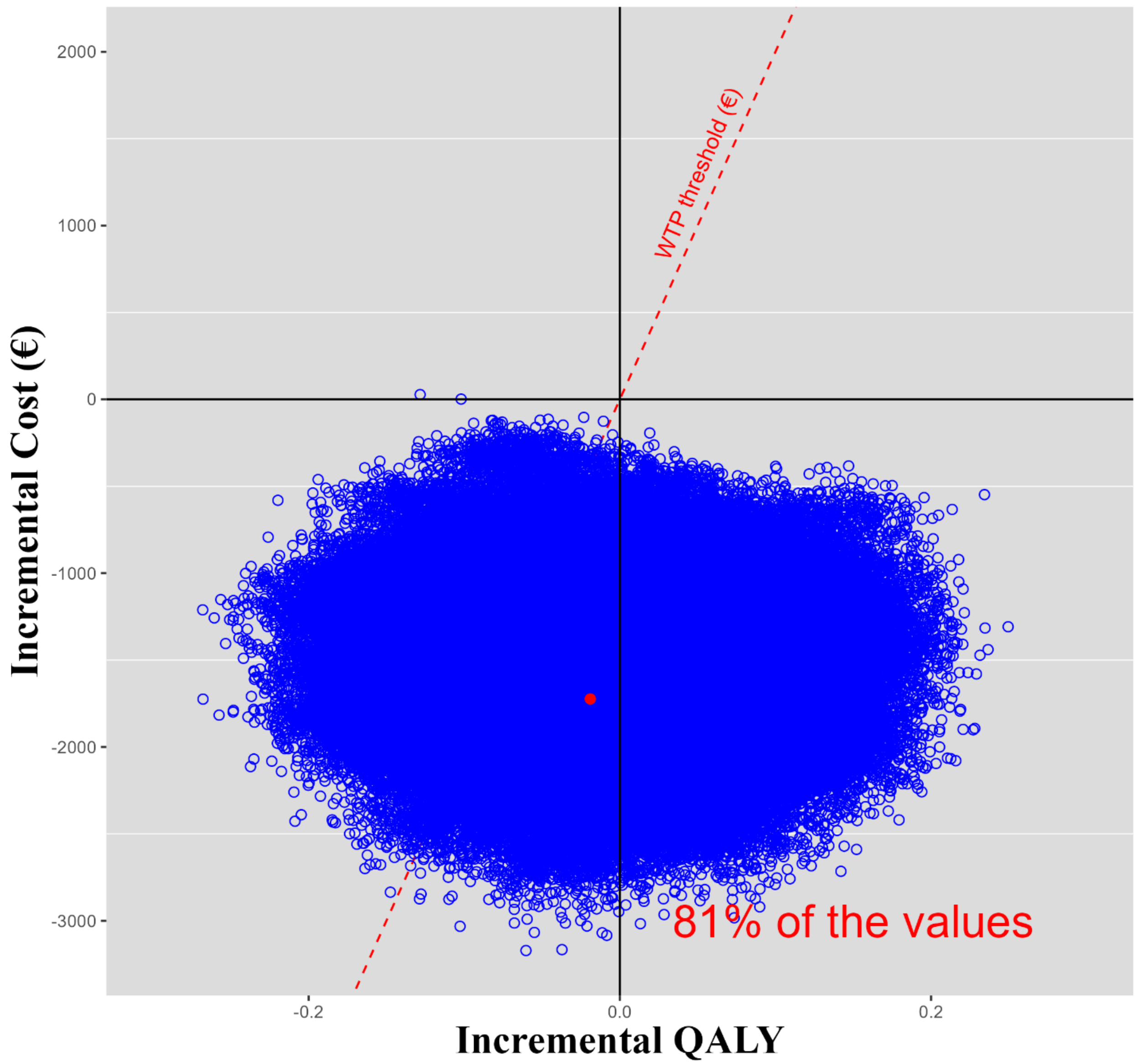


**Fig S2C** Cost-effectiveness plane at three-year follow-up

Probabilistic sensitivity analyses of cost-effectiveness at the three-year follow-up performed by 1000 bootstraps for each imputed dataset (500,000 circles in total), which depicts the uncertainty surrounding the ICER (red) point.

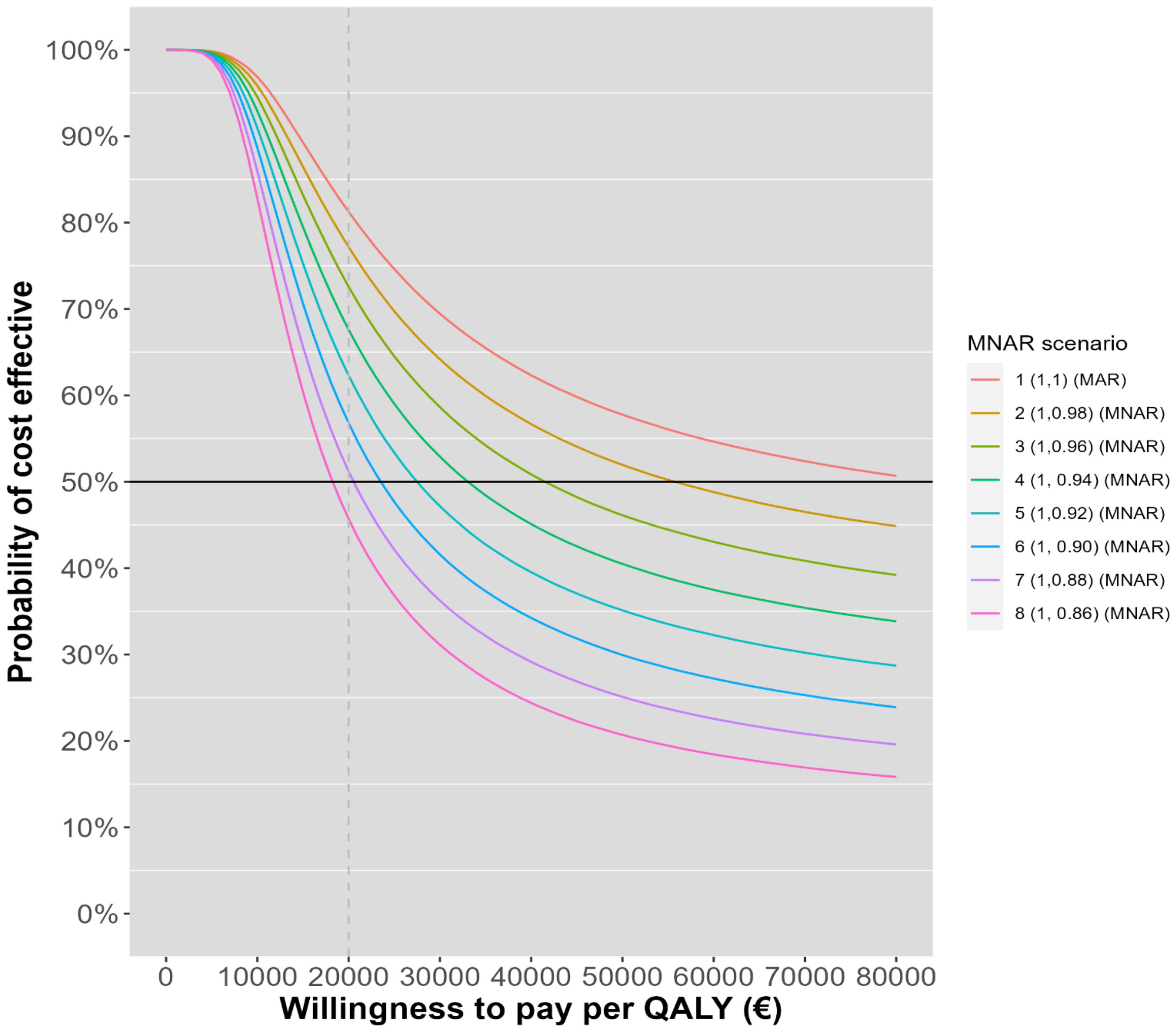


MAR= missing at random, MNAR= missing not at random

**Fig S3** Dual approach combining pattern mixture modelling with tipping point analysis at three-year follow-up

Pattern mixture modelling used a 2% delta, while tipping point analysis increased this delta systematically in 2% increments. Each step (from 2 to 7) decreases the QALY of missing data in the CT group by 2% compared to the observed CT QALY values. The threshold or 'tipping point' was reached when the cost acceptability curve descended below a 50% probability at a willingness-to-pay (WTP) threshold of €20,000/QALY at three-year follow-up. The figure shows that the missing QALY in the CT group has to be at least 14% lower than the observed QALY to make ICA more cost-effective at a WTP threshold of €20,000/QALY.

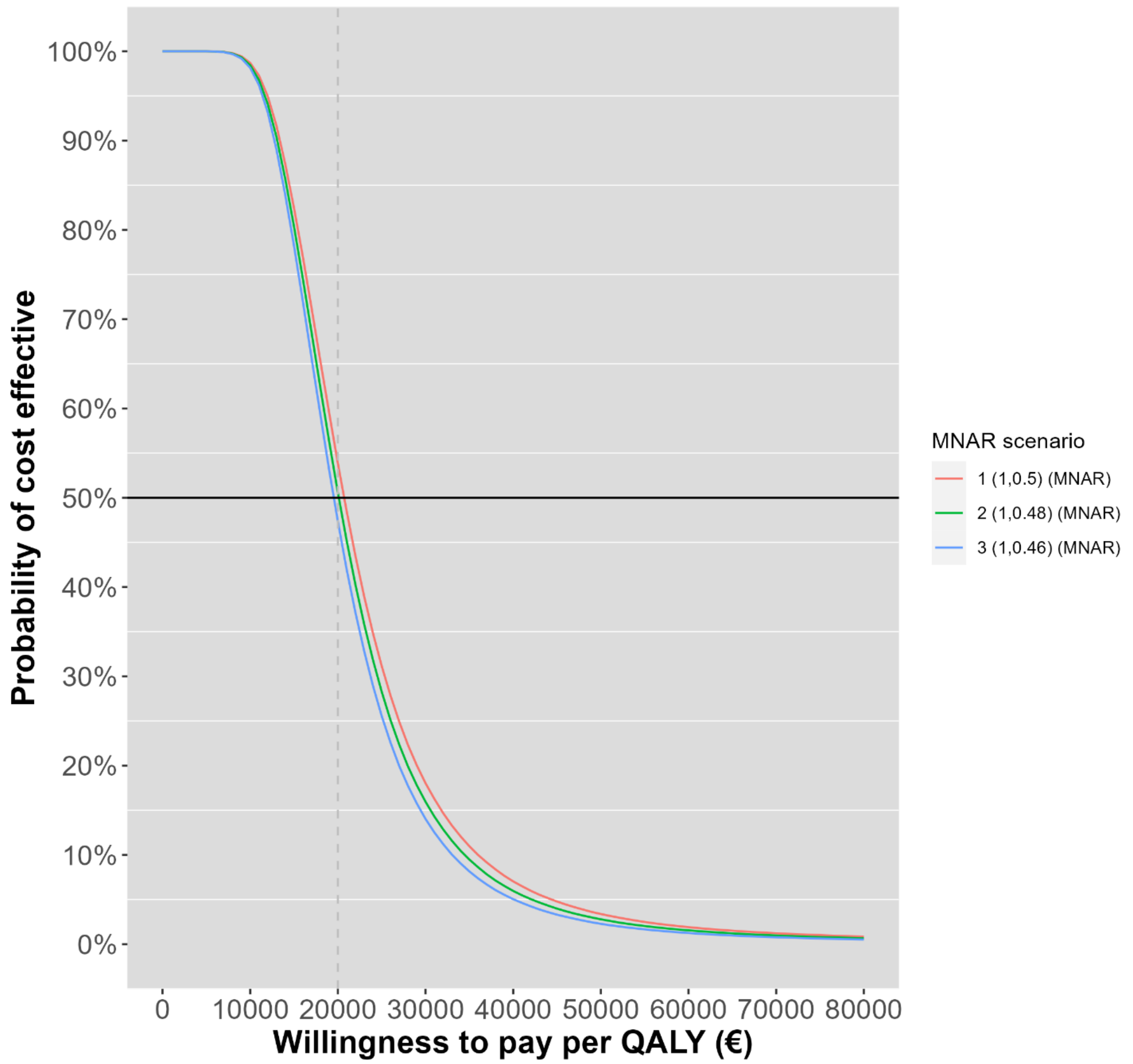


**Fig S4** Dual approach combining pattern mixture modelling with tipping point analysis at one-year follow-up

A dual approach combining pattern mixture modelling with tipping point analysis was used. Pattern mixture modelling used a 2% delta, while the tipping point analysis increased this delta systematically in 2% increments. Each step decreased the QALY of missing data in the CT group by 2% compared to the observed CT QALY values. The threshold or tipping point was reached when the cost acceptability curve descended below a 50% probability at a willingness-to-pay (WTP) threshold of €20,000/QALY at three-year follow-up. For simplicity, the graph starts at 50% delta and shows that the percentage of missing QALYs in the CT group has to be at least 54% lower than the observed QALY value to make ICA more cost-effective at a WTP threshold of €20,000/QALY.

**Table S1 Reported** DRG codings, DRG texts and reimbursed costs at baseline in the two groups

| DRG coding | DRG text | CT group (n=167) | CT group cost in € median (95% CI) | ICA group (n=162) | ICA group cost in € median (95% CI) |
|---|---|---|---|---|---|
| F66B | Coronary arteriosclerosis without extremely severe complication or comorbidity | 18 (10.8%) | 861 (605-1375) | 30 (18.5%) | 1781 (1683-1924) |
| F49G | Invasive cardiological diagnostics except for acute myocardial infarction, one day of occupancy, age> 14 years, without specific intervention | 3 (1.8%) | 2011 | 43 (26.5%) | 1866 (1679-1923) |
| F49E | Invasive cardiological diagnostics except for acute myocardial infarction, more than 2 days of occupancy, without extremely severe complication or comorbidity, age> 14 years, without cardiac mapping, without complex intervention | 5 (3.0%) | 2421(2421-3190) | 34 (21.0%) | 2474 (2238-2808) |
| F58B | Percutaneous coronary angioplasty without extremely severe complication or comorbidity | 8 (4.8%) | 2968 (2850-4457) | 16 (9.9%) | 2759 (2454-3258) |
| F71B | Non-severe cardiac arrhythmia and conduction disorders without extremely severe complication or comorbidity, without catheter-assisted electrophysiological examination of the heart | 11 (6.6%) | 881 (692-1610) | 4 (2.5%) | 1944 (1201-2067) |
| F56B | Percutaneous coronary angioplasty with highly complex intervention, without extremely severe complication or comorbidity, or cryoplasty | 4 (2.4%) | 5094 (3781-5637) | 5 (3.1%) | 4223 (3174-4369) |
| F67D | Hypertension without specific endocrine gland disease, without extremely severe or severe complication or comorbidity, age> 15 years | 5 (3.0%) | 1627 (1351-2128) | 4 (2.5%) | 1551 (1251-1965) |
| F74Z | Chest pain | 9 (5.4%)) | 1183 (512-1503) | 3 (1.9%) | 1525 (1247-1607) |
| F49F | Invasive cardiological diagnostics except for acute myocardial infarction, one day of occupancy, age> 14 years, with a specific intervention | 2 (1.2%) | 2987 (2986-2986) | 5 (3.1%) | 2420 (2385-3504) |
| F72B | Unstable angina without extremely severe complication or comorbidity | 6 (3.6%) | 1551 (1390-1615) | 2 (1.2%) | 2238 (2058-2418) |
| F75D | Other diseases of the circulatory system without extremely severe complication or comorbidity, age> 17 years | 1 (0.6%) | 2391 | 2 (1.2%) | 1712 (1707-1718) |
| F57Z | Percutaneous coronary angioplasty with complex intervention without extremely severe complication or comorbidity | 1 (0.6%) | 3756 | 1 (0.6%) | 3765 |

| | | | | | |
|---|---|---|---|---|---|
| F71C | Non-severe cardiac arrhythmia and conduction disorders without extremely severe or severe complication or comorbidity | 1 (0.6%) | 1443 | 1 (0.6%) | 1212 |
| F72C | Unstable angina without extremely severe or severe complication or comorbidity | 0 | n/a | 2 (1.2%) | 1549 |
| F06E | Coronary bypass surgery without multiple complex OR procedures, without complicating constellations, without carotid surgery, with invasive cardiological diagnostics or with intraoperative ablation, except in the case of infarction, without reoperation | 0 | n/a | 1 (0.6%) | 14681 |
| F49C | Invasive cardiological diagnostics except for acute myocardial infarction, less than 3 days of occupancy or more than 2 days of occupancy without express. severe complication or comorbidity, age <15 years or more than 2 days of occupancy, without express. black complication or comorbidity, age> 14 years, with cardiac mapping | 1 (0.6%) | 3167 | 0 | n/a |
| F49D | Invasive cardiological diagnostics except for acute myocardial infarction, more than 2 days of occupancy, without extremely severe complication or comorbidity, age> 14 years, without cardiac mapping, with complex intervention | 0 | n/a | 1 (0.6%) | 3802 |
| F52B | Percutaneous coronary angioplasty with complex diagnosis, without extremely severe complication or comorbidity or with intracoronary brachytherapy | 0 | n/a | 1 (0.6%) | 5359 |
| F54Z | Complex or multiple vascular interventions without complications. Constellation, without revision, without complications. Diagnostics, age> 2 years, without certain bilateral vascular interventions or moderately complication.<br>Vascular interventions, without severe complication or comorbidity, without rotational thrombectomy. | 0 | n/a | 1 (0.6%) | 4020 |
| F62B | Heart failure and shock without extremely severe complication or comorbidity or without dialysis, without resuscitation, without complicating diagnosis | 0 | n/a | 1 (0.6%) | 2079 |
| F62C | Heart failure and shock without severe complication or comorbidity | 0 | n/a | 1 (0.6%) | 1834 |
| F68B | Heart failure and shock without extremely severe complication or comorbidity | 1 (0.6%) | 2810 | 0 | |

| | | | | | |
|---|---|---|---|---|---|
| F69B | Valvular heart disease without extremely severe or severe complication or comorbidity | 0 | n/a | 1 (0.6%) | 1824 |
| I24.4 | Other acute ischaemic heart disease | 1 (0.6%) | 4321 | 0 | n/a |
| n/a | Outpatient* (DRG text n/a) | 90 (53.9%) | 185 (185-187) | 2 (1.2%) | 643 |
| Total | | 167 | 187 (187-691) | 162 | 2030 (1892-2106) |

*Cost data collection at baseline for inpatients: at baseline, information on amount reimbursed to the hospital for patients who stayed as inpatients for their examinations was obtained from the billing information recorded at Charité using the coronary DRG (diagnosis-related group; http://www.g-drg.de/) of each patient if possible. Whenever information on actual reimbursement was not available (16 patients: 15 in CT group; 1 in ICA group) or non coronary DRG reimbursement occurred for DRGs (19 (CT: 8; ICA: 11), DRG grouping costs were used instead and calculated with the calculator of the German DRG Research Group (www.drg-reseach-group.de). The required ICD (International Statistical Classification of Diseases and Related Health Problems) codes, OPS (Operation and Procedure Code) codes and length of individual hospital stay were collected for each patient from the hospital billing system. For calculation of hospital payment, the DRG cost weight was multiplied with a uniform state-wide base rate, which was adjusted for each year and the state of Berlin (InEK GmbH). Cost data collection at baseline for outpatients: costs for outpatient examinations were determined from the reimbursement rates according to the Einheitlicher Bewertungsmaßstab (EBM; www.kbv.de). The following EBM reimbursement numbers were used for CT examinations: 34330, 34345, and 24211 for patients aged ≤59 years and 24212 for patients aged >59 years. The reimbursement numbers used for ICA were 1520 and 34291.

**Table S2** Reported DRG codings, DRG texts and reimbursed costs for cardiovascular-related hospitalisations at follow-up (until 3 years) in the two groups

| DRG coding | DRG text | CT group (n=167) | CT group Cost in € median (95% CI) | ICA group (n=162) | ICA group Cost in € median (95% CI) |
|---|---|---|---|---|---|
| **Hospitalisations due to chest pain, invasive cardiological diagnosis, angina pectoris, revascularisation and acute myocardial infarction** | | **26** | | **20** | |
| F41B | Invasive cardiological diagnostics in acute myocardial infarction without extremely severe complication or comorbidity | 1 | 3265 (n/a) | 2 | 2760 (1595-3924) |
| F49D | Invasive cardiological diagnostics except for acute myocardial infarction, without extremely severe complication or comorbidity, expenditure points, age> 17 years, with severe complication or comorbidity, more than one day of occupancy | 1 | 3626 (n/a) | 0 | (n/a) |
| F49E | Invasive cardiological diagnostics except for acute myocardial infarction, more than 2 days of occupancy, without extremely severe complication or comorbidity, age> 14 years, without cardiac mapping, without complex surgery | 3 | 2371 (0-2374) | 0 | (n/a) |
| F49F | Invasive cardiological diagnosis except for acute myocardial infarction, without extremely severe complication or comorbidity, age> 14 years, without cardiac mapping, without severe complication or comorbidity, without complex diagnosis, with specific intervention | 3 | 2774 | 0 | n/a |
| F49G | Invasive cardiological diagnosis except for acute myocardial infarction, without extremely severe complication or comorbidity, age> 14 years, without cardiac mapping, without severe complication or comorbidity, without complex diagnosis, without specific intervention | 5 | 2122 (2022-2128) | 2 | 2022 (2011-2122) |
| F56B | Percutaneous coronary angioplasty with highly complex intervention, without certain highly complex intervention, without extremely severe complication or comorbidity, or cryoplasty | 1 | 3710 | 3 | 3680 (3615-4059) |
| F57Z | Percutaneous coronary angioplasty with complex intervention without extremely severe complication or comorbidity | 2 | 2739 (2712-2766) | 1 | 2766 |
| F58B | Percutaneous coronary angioplasty without extremely severe complication or comorbidity | 2 | 2921 (2884-2958) | 4 | 2842 (2758-2884) |
| F60B | Acute myocardial infarction without invasive cardiological diagnostics without extremely severe complication or comorbidity | 0 | | 1 | 765 |

| | | | | | |
|---|---|---|---|---|---|
| F66B | Coronary arteriosclerosis without extremely severe complication or comorbidity | 5 | 1417 (1417-1509 | 4 | 1411 (1405-1417) |
| F67D | Invasive cardiological diagnosis except for acute myocardial infarction, without extremely severe complication or comorbidity, age> 14 years, without cardiac mapping, without severe complication or comorbidity, without complex diagnosis, with specific intervention | 1 | 570 | 1 | 1471 |
| F72B | Unstable angina without extremely severe complication or comorbidity | 1 | 620 | 0 | n/a |
| F74Z | Chest pain and other and unspecified diseases of the circulatory system | 0 | n/a | 2 | 966 (558-1374) |
| F06B | Coronary bypass surgery without invasive cardiological diagnostics, without complicating constellations, without carotid intervention, without intraoperative ablation | 1 | 10988 | 0 | n/a |
| **Hospitalisations due to arrhythmia, tachyarrhythmia and pacemaker implantation/revision** | | **5** | | **19** | |
| F12G | Implantation of a pacemaker, two-chamber system, without complex surgery, age> 15 years, without probe removal with an excimer laser, without extremely severe complication or comorbidity, or isolated open surgical probe implantation | 0 | n/a | 3 | 5146 (5144-51471) |
| F50A | Ablative measures for tachyarrhythmia with complex ablation in the left atrium or highly complex ablation or implantation of an event recorder | 1 | 8687 | 4 | 8432 (8400-8432) |
| F50B | Ablative measures for tachyarrhythmias without complex ablation in the left atrium, without highly complex ablation, without implantation of an event recorder, with transseptal left heart catheter examination | 0 | n/a | 1 | 6081 |
| F50C | Ablative measures for tachyarrhythmias with complex ablation, without complex ablation in the left atrium, without highly complex ablation, without implantation of an event recorder, without transseptal left heart catheter examination | 0 | n/a | 5 | 6357 (5652-6357) |
| F50D | Ablative measures for tachyarrhythmias without complex ablation, without complicating surgery, age> 15 years | 1 | 4239 | 2 | 4152 (4066-4239) |
| F71A | Non-severe cardiac arrhythmia and conduction disorders with extremely severe complication or comorbidity | 1 | 4160 | 0 | |
| F71B | Non-severe cardiac arrhythmia and conduction disorders without extremely severe complication or comorbidity, without catheter-assisted electrophysiological examination of the heart | 1 | 1665 | 2 | 1553 (1553-1568) |

| | | | | | |
|---|---|---|---|---|---|
| F71C | Non-severe cardiac arrhythmia and conduction disorders without extremely severe or severe complication or comorbidity | 1 | 1881 | 2 | 1454 (1443-1464) |
| | **Other cardiac-related hospitalisations** | **8** | | **5** | |
| B69C | Transient ischaemic attack (TIA) and extracranial vascular occlusion with neurological complex treatment of acute stroke without extremely severe complication or comorbidity or with other neurological complex treatment or with extremely severe complication or comorbidity | 0 | | 1 | 3232 |
| E63Z | Sleep apnea syndrome | 3 | 820 (799-842) | 2 | 766 (766-766) |
| E64C | Respiratory failure, more than one day of occupancy, without extremely severe complication or comorbidity, age> 9 years | 0 | n/a | 1 | 2740 |
| E69E | Bronchitis and bronchial asthma, ages> 5 years and <16 years, an occupancy day or without extremely heavy or severe complication or comorbidity or breathing discomfort and symptoms without complex diagnosis, age <16 years | 2 | 1472 (1453-1490) | 0 | |
| F19C | Other percutaneous transluminal intervention on the heart, aorta and pulmonary vessels without extremely severe complication or comorbidity, age> 5 years | 1 | 4957 | 1 | 5349 |
| F62C | Heart failure and shock without extremely severe complication or comorbidity | 1 | 2401 | 0 | |
| F75D | Other diseases of the circulatory system without extremely severe complication or comorbidity, age> 17 years | 1 | 2083 | 0 | 0 |
| **Total cardiac-related hospitalisations** | | **39** | | **44** | |

* Cost data collection at follow-up for inpatients: for inpatient procedures, DRG grouping costs were used and calculated with the calculator of the German DRG Research Group (diagnosis-related group; http://www.g-drg.de/). The required ICD (International Statistical Classification of Diseases and Related Health Problems) codes, OPS (Operation and Procedure Code) codes and length of individual hospital stay were collected for each patient from the hospital billing system. For calculation of hospital payment, the DRG cost weights were multiplied with a uniform state-wide base rate, which was adjusted for each year and the state of Berlin (InEK GmbH).

**Table S3 EBM** numbers and costs of outpatient healthcare services

| Test | EBM | Additional costs | EBM (2009-2012) | EBM (2013-2018) | Costs § (year 2017) |
|---|---|---|---|---|---|
| Ex-ECG | 13251; 1600 | n/a | 565;110 | 200;39 | 25.17 |
| Dobutamine stress ECHO | 33031;1600;2100 | €2.19 dobutamine and atropine | 2360;110;160 | 835;39;57 | 99.35 |
| Dobutamine stress MRI | 34430; (24211;24212)† | €2.19 dobutamine and atropine | 3430;125;150 | 1213;44;53 | 189.20 |
| MSCT coronary angiography | 34330;34345; (24211;24212)† | €51.26 non-ionic iodinated contrast agent | 1865;645;125;150 | 660;228;44,53 | 143.82 |
| ICA | 1520;34291 | €51.26 non-ionic iodinated contrast agent | 2720;8865 | 962;3135 | 482.67 |
| Outpatient cardiologist visits | 13545 | n/a | 1920 | 679 | 71.50 |
| Myocardial scintigraphy | 17330 (24211;24212)† | €77.84 Technetium | 2720;125;150 | 962;39;44,53 | 193.46 |
| Emergency department visits | 1412 | n/a | 1770 | 626 | 65.92 |

* Cost data collection at follow-up for outpatients: costs for outpatient services were determined from the reimbursement rates according to the Einheitlicher Bewertungsmaßstab (EBM; www.kbv.de).

† EBM code 24211 for patients aged ≤59 year and 24212 for patients aged >59

‡https://www.deutschlandfunkkultur.de/reha-massnahmen-als-wirtschaftsfaktor-der-preis-der.976.de.html?dram:article_id=345740

§ For illustration, costs are provided for 2017. For the calculation of the costs per patient, adjustments were made for each year.

**Table S4** Average cost in € per DDD (defined daily dose) for each year

| Agent | Average cost in € per DDD (defined daily dose) according to the Arzneiverordnungs-Report 2009-2018 (www.wido.de) * | | | | | | | | | |
|---|---|---|---|---|---|---|---|---|---|---|
| | 2009 | 2010 | 2011 | 2012 | 2013 | 2014 | 2015 | 2016 | 2017 | 2018 |
| **Lipid-lowering agents** | | | | | | | | | | |
| Simvastatin | 0.33 | 0.29 | 0.27 | 0.22 | 0.21 | 0.2 | 0.19 | 0.19 | 0.19 | 0.19 |
| Pravastatin | 0.44 | 0.4 | 0.34 | 0.25 | 0.24 | 0.23 | 0.22 | 0.21 | 0.21 | 0.21 |
| Fluvastatin | 0.53 | 0.39 | 0.36 | 0.28 | 0.26 | 0.25 | 0.24 | 0.23 | 0.23 | 0.23 |
| Atorvastatin | 1.07 | 1.08 | 1 | 1 | 0.23 | 0.15 | 0.14 | 0.13 | 0.13 | 0.13 |
| Ezetrol | 2.04 | 2.09 | 2.12 | 1.82 | 1.8 | 1.8 | 1.92 | 1.72 | 1.72 | 1.71 |
| Bezafibrat | 0.45 | 0.44 | 0.46 | 0.4 | 0.4 | 0.4 | 0.4 | 0.4 | 0.41 | 0.41 |
| **Antianginal agents** | | | | | | | | | | |
| Beta-blockers | | | | | | | | | | |
| Atenolol | 0.46 | 0.46 | 0.46 | 0.41 | 0.45 | 0.44 | 0.43 | 0.23 | 0.23 | 0.23 |
| Bisoprolol | 0.34 | 0.32 | 0.25 | 0.22 | 0.25 | 0.24 | 0.24 | 0.25 | 0.25 | 0.26 |
| Carvedilol | 0.46 | 0.45 | 0.43 | 0.37 | 0.35 | 0.35 | 0.36 | 0.37 | 0.37 | 0.37 |
| Metoprolol | 0.33 | 0.33 | 0.33 | 0.29 | 0.29 | 0.29 | 0.29 | 0.28 | 0.28 | 0.28 |
| Mobloc | 0.25 | 0.18 | 0.22 | 0.18 | 0.16 | 0.14 | 0.14 | 0.13 | 0.13 | 0.13 |
| Nebivolol | 0.61 | 0.52 | 0.51 | 0.48 | 0.19 | 0.23 | 0.18 | 0.13 | 0.13 | 0.13 |
| Propranolol | 0.8 | 0.8 | 0.8 | 0.79 | 0.69 | 0.71 | 0.76 | 0.77 | 0.77 | 0.77 |
| Calcium-channel blockers | | | | | | | | | | |
| Amlodipine | 0.12 | 0.11 | 0.1 | 0.08 | 0.08 | 0.08 | 0.09 | 0.09 | 0.09 | 0.09 |
| Lercanidipine | 0.18 | 0.17 | 0.17 | 0.15 | 0.15 | 0.16 | 0.16 | 0.16 | 0.1 | 0.09 |
| Felodipine | 0.32 | 0.32 | 0.32 | 0.3 | 0.3 | 0.29 | 0.28 | 0.29 | 0.3 | 0.3 |
| Nifedipine | 0.71 | 0.29 | 0.3 | 0.25 | 0.26 | 0.27 | 0.29 | 0.29 | 0.3 | 0.3 |
| Nitrendipine | 0.19 | 0.19 | 0.2 | 0.17 | 0.17 | 0.18 | 0.19 | 0.2 | 0.2 | 0.21 |
| Indapamide | 0.47 | 0.45 | 0.44 | 0.37 | 0.37 | 0.36 | 0.37 | 0.37 | 0.37 | 0.37 |
| Nitrates | | | | | | | | | | |
| Isosorbide dinitrate | 0.24 | 0.24 | 0.24 | 0.21 | 0.22 | 0.23 | 0.23 | 0.24 | 0.23 | 0.23 |
| Other antianginals | | | | | | | | | | |
| Amiodarone | 0.74 | 0.72 | 0.7 | 0.64 | 0.64 | 0.64 | 0.62 | 0.62 | 0.58 | 0.52 |
| Ranolazine | n/a | n/a | 4.34 | 3.74 | 3.75 | 3.77 | 4.05 | 4.05 | 4.01 | 4.04 |
| Diltiazem | 0.49 | 0.48 | 0.48 | 0.42 | 0.44 | 0.45 | 0.45 | 0.44 | 0.44 | 0.44 |
| Ivabradine | 1.62 | 1.74 | 1.74 | 1.94 | 2.02 | 2.03 | 2.19 | 2.18 | 2.19 | 2.05 |
| Flecainide | 1.28 | 1.17 | 1.02 | 0.9 | 0.89 | 0.88 | 0.89 | 0.87 | 0.86 | 0.86 |
| Digitoxin | 0.2 | 0.21 | 0.21 | 0.17 | 0.18 | 0.19 | 0.22 | 0.19 | 0.19 | 0.19 |
| **Antiplatelet agents** | | | | | | | | | | |
| Acetylsalicylic acid | 0.04 | 0.12 | 0.11 | 0.11 | 0.12 | 0.14 | 0.16 | 0.18 | 0.2 | 0.2 |
| Clopidogrel | 2.55 | 2.34 | 1.88 | 0.96 | 0.79 | 0.61 | 0.52 | 0.37 | 0.34 | 0.32 |
| Pravix | 2.61 | 2.8 | 2.76 | 2.35 | 2.3 | 2.2 | 2.26 | 0.82 | 0.46 | 0.46 |
| **Antihypertensive agents** | | | | | | | | | | |
| ACE | | | | | | | | | | |
| Enalapril | 0.76 | 0.13 | 0.12 | 0.09 | 0.09 | 0.1 | 0.1 | 0.1 | 0.1 | 0.09 |
| Benazepril | 0.14 | 0.12 | 0.11 | 0.09 | 0.09 | 0.09 | 0.12 | 0.11 | 0.09 | 0.09 |
| Captopril | 0.18 | 0.18 | 0.17 | 0.13 | 0.14 | 0.14 | 0.15 | 0.15 | 0.14 | 0.14 |

| | | | | | | | | | | |
|---|---|---|---|---|---|---|---|---|---|---|
| Ramipril | 0.07 | 0.06 | 0.06 | 0.05 | 0.05 | 0.05 | 0.06 | 0.06 | 0.06 | 0.06 |
| Lisinopril | 0.14 | 0.13 | 0.12 | 0.1 | 0.1 | 0.1 | 0.1 | 0.1 | 0.1 | 0.1 |
| Verapamil | 0.64 | 0.37 | 0.37 | 0.32 | 0.32 | 0.33 | 0.33 | 0.33 | 0.33 | 0.33 |
| ARB | | | | | | | | | | |
| Telmisartan | 0.61 | 0.62 | 0.62 | 0.61 | 0.6 | 0.6 | 0.35 | 0.19 | 0.19 | 0.19 |
| Valsartan | 0.61 | 0.52 | 0.51 | 0.48 | 0.19 | 0.23 | 0.18 | 0.13 | 0.13 | 0.13 |
| Candesartan | 0.49 | 0.47 | 0.47 | 0.45 | 0.31 | 0.2 | 0.16 | 0.13 | 0.12 | 0.12 |
| Azilsartan medoxomil | n/a | n/a | n/a | n/a | 0.77 | 0.78 | n/a | n/a | n/a | n/a |
| Irbesartan | 0.7 | 0.7 | 0.7 | 0.67 | 0.65 | 0.43 | 0.3 | 0.21 | 0.21 | 0.2 |
| Eprosartan | 0.9 | 0.77 | 0.88 | 0.69 | 0.68 | 0.69 | 0.57 | 0.47 | 0.29 | 0.27 |
| Atacand | 1.07 | 1.08 | 1.11 | 1.09 | 0.79 | 0.51 | 0.44 | 0.34 | 0.26 | 0.27 |
| Losartan | 0.87 | 0.84 | 0.58 | 0.31 | 0.27 | 0.26 | 0.23 | 0.21 | 0.21 | 0.19 |
| Olmesartan | 0.76 | 0.88 | 1.08 | 0.87 | 0.85 | 0.85 | 0.85 | 0.86 | 0.86 | 0.86 |
| **Diuretics** | | | | | | | | | | |
| Amiloride | 0.15 | 0.15 | 0.15 | 0.12 | 0.14 | 0.16 | 0.17 | 0.18 | 0.18 | 0.21 |
| Furosemide | 0.13 | 0.13 | 0.13 | 0.11 | 0.11 | 0.11 | 0.12 | 0.12 | 0.12 | 0.12 |
| Hydrochlorothiazide | 0.19 | 0.19 | 0.18 | 0.16 | 0.16 | 0.17 | 0.17 | 0.17 | 0.18 | 0.18 |
| Indapamide | 0.47 | 0.47 | 0.45 | 0.44 | 0.37 | 0.37 | 0.36 | 0.37 | 0.37 | 0.37 |
| Spironolactone | 0.38 | 0.35 | 0.34 | 0.3 | 0.3 | 0.3 | 0.31 | 0.31 | 0.31 | 0.31 |
| Spironothiazide | 0.37 | 0.37 | 0.37 | 0.33 | 0.32 | 0.31 | 0.32 | 0.32 | 0.32 | 0.32 |
| Torasemide | 0.36 | 0.21 | 0.19 | 0.16 | 0.16 | 0.17 | 0.17 | 0.18 | 0.18 | 0.18 |
| Triamterene | 0.18 | 0.18 | 0.18 | 0.15 | 0.14 | 0.15 | 0.15 | 0.22 | 0.21 | 0.21 |
| Xipamide | 0.17 | 0.16 | 0.17 | 0.14 | 0.15 | 0.16 | 0.16 | 0.17 | 0.17 | 0.18 |

* Cost data collection at follow-up for medications: average costs per DDD (defined daily dose) were taken from the Arzneiverordnungs-Report 2009-2018 (www.wido.de) and used for calculating medication costs. Average costs per DDD were cumulated between randomisation date and the last follow-up date.

**Table S5 Comparison** of patient-reported outcomes at baseline and at the three follow-up time points (median follow-up of 3.7 years).

| | Baseline | | One-year follow-up | | Two-year follow-up | | Three-year follow-up | |
|---|---|---|---|---|---|---|---|---|
| **PROMs** | **CT group** OR (95% CI) | **ICA group** OR (95% CI) | **CT group** OR (95% CI) | **ICA group** OR (95% CI) | **CT group** OR (95% CI) | **ICA group** OR (95% CI) | **CT group** OR (95% CI) | **ICA group** OR (95% CI) |
| **EQ-5D-3L** | | | | | | | | |
| Utilities* | 0.70 (0.66- | 0.73 (0.69- | 0.74 (0.69- | 0.77 (0.72- | 0.82 (0.78- | 0.81 (0.77- | 0.78 (0.73- | 0.81 (0.75-0.87) |
| EQ VAS† | 66.1 (63.2- | 63.9 (60.8- | 66.2 (62.7- | 68.6 (65.0- | 67.8 (64.6- | 70.6 (67.4- | 68.8 (64.4- | 69.6 (65.1-74.2) |
| Data missing | 12/167 (7.2%) | 20/162 (12.3%) | 33/167 (19.8%) | 34/162 (21.0%) | 31/167 (18.6%) | 47/162 (29.0%) | 44/167 (26.3%) | 45/162 (27.8%) |
| **MacNew‡** | | | | | | | | |
| Physical | 5.2 (5.0-5.4) | 5.3 (5.2-5.5) | 5.8 (5.7-6.0) | 5.8 (5.7-6.0) | 5.7 (5.6-5.9) | 6.0 (5.8-6.1) | 6.0 (5.8-6.3) | 6.0 (5.8-6.3) |
| Emotional | 5.0 (4.9-5.2) | 5.0 (4.9-5.2) | 5.1 (4.9-5.3) | 5.2 (5.0-5.4) | 5.1 (4.9-5.3) | 5.3 (5.1-5.5) | 5.3 (5.1-5.5) | 5.4 (5.2-5.7) |
| Social | 5.6 (5.4-5.7) | 5.6 (5.4-5.7) | 5.5 (5.4-5.7) | 5.7 (5.5-5.9) | 5.5 (5.3-5.7) | 5.8 (5.6-5.9) | 5.7 (5.5-5.9) | 5.8 (5.6-5.9) |
| Global | 5.2 (5.0-5.3) | 5.2 (5.1-5.4) | 5.4 (5.2-5.6) | 5.5 (5.3-5.6) | 5.3 (5.2-5.5) | 5.5 (5.4-5.7) | 5.6 (5.4-5.8) | 5.7 (5.5-5.9) |
| Data missing | 5/167 (3.0%) | 16/162 (9.9%) | 31/167 (18.6%) | 32/162 (19.8%) | 31/167 (19.8%) | 47/162 (29.0%) | 44/167 (26.3%) | 47/162 (29.0%) |
| **HADS§** | | | | | | | | |
| Depression | 5.1 (4.5-5.6) | 5.2 (4.6-5.8) | 5.4 (4.8-6.1) | 5.6 (4.9-6.2) | 5.6 (5.0-6.2) | 5.2 (4.6-5.9) | 5.1 (4.3-6.0) | 4.8 (4.0-5.7) |
| Anxiety | 6.5 (5.9-7.1) | 6.9 (6.3-7.6) | 6.4 (5.8-7.1) | 6.3 (5.6-7.0) | 6.5 (5.8-7.1) | 6.8 (6.1-7.4) | 6.1 (5.2-6.9) | 6.3 (5.4-7.2) |
| Data missing | 6/167 (3.6%) | 20/162 (12.3%) | 33/167 (19.8%) | 34/162 (21.0%) | 34/167 (20.4%) | 49/162 (30.2%) | 45/167 (26.9%) | 49/162 (30.2%) |

CT = computed tomography; ICA = invasive coronary angiography; EQ-5D-3L = Quality of life–5 dimensions; CI = confidence interval; VAS = visual-analogue scale; MacNew = heart disease health-related quality of life; HADS= Hospital Anxiety and Depression Scale; PROMs = patient-reported outcome measures; OR =odds ratio;
* We used five-dimensional German value sets for the utility scores calculation.
† EQ VAS scale ranges from 0 to 100, with higher scores indicating better health status.
‡The maximum possible score in any domain is 7. Higher scores indicate better patient condition.
§ Utility scores were calculated using the German tariff. HADS Depression and Anxiety (0-21). Higher scores indicate more severe depression or anxiety.

**Table S6** Comparison of randomised trials (including at least one group undergoing CT) investigating costs in patients with stable chest pain or suspected coronary artery disease

| Trial name | Year | Comparator | Time horizon | Method | Results | Reference |
|---|---|---|---|---|---|---|
| CAD-Man | 2023 | CT vs. ICA | 3 years | Cost-effectiveness analysis | CT vs. ICA:<br>€995.8 (799.1 to 1197.4) v €2305.8 (2149.3 to 2563.4) --> Cost/patient (95% CI) index test cost<br>€1879.8 (1532.3 to 2292.4) v €3527.6 (3120.8 to 3996.9) --> Cost/patient (95% CI) costs up to 1 year<br>€2291.8 (1881.3 to 2790.2) v €3900.2 (3446.5to 4421.6) --> Cost/patient (95% CI) costs up to 2 years<br>€2582.0 (2133.1 to 3128.3) v €4125.297 (€3639.0 to 4701.9) --> Cost/patient (95% CI) costs up to 3 years | Current study |
| CONSERVE | 2019 | CT vs. ICA | 1 year | Modelled diagnostic cost using a hybrid approach in which utilization data were multiplied by published cost estimates, including Medicare payment rates for fiscal year 2016 | CT vs. ICA:<br>($401 vs. $2549) --> per-patient index test cost<br>($1183 vs. $2755) --> per-patient total diagnostic cost (1 year) | Chang HJ et al[1] |
| CAT-CAD | 2020 | CT vs. ICA | 90 days | Calculation of cumulative cost for each strategy by multiplying the number of resources by standardized costs according to medical databases and the 2015 Procedural Reimbursement Payment Guide. | CT v ICA:<br>($836 vs. $2,284) --> per patient costs for CAD diagnosis;<br>($4630 vs. $8,380) --> per patient costs for diagnostic and therapeutic care (90 days); | Rudziński PN et al[2] |
| RAPID-CTCA RCT | 2022 | Early CT supplementing standard care vs. standard care alone | 1 year | Cost-effectiveness analysis | Early CT supplementing standard care v standard care alone:<br>£7414.13 (£6840.20, £7988.07) v £6845.11 (£6337.42, £7352.79) --> mean total health-care costs (1 year)<br><br>QALYs<br>(0.7488 (0.7353, 0.7621) v 0.7577 (0.7456, 0.7699), mean difference -0.009, 95% CI -0.026 to 0.010; p=0.377) | Thokala P et al[3] |
| n/a | 2007 | MSCT alone vs. standard diagnostic evaluation | Costs during the index ED visit | To derive the ED cost of care, total patient charges in the ED calculated by the hospital billing department were multiplied by the hospital's ED cost-to-charge ratio. | MSCT alone vs. standard diagnostic evaluation:<br>$1,586 v $1,872 | Goldstein JA et al[4] |
| PROMISE trial | 2016 | CT vs. functional testing | 3 years | Technical costs of the initial (outpatient) testing strategy were estimated from Premier Research Database data. Hospital-based costs were estimated using hospital bills and Medicare cost-charge ratios. Physician fees were taken from the Medicare Physician Fee Schedule. | CT vs. functional testing<br>$2494vs. $2240 → at 90 days<br>The mean cost difference between the groups over 3 years remained small. | Mark DB et al[5] |

| | | | | | | |
|---|---|---|---|---|---|---|
| CRESCENT trial | 2016 | CT vs. functional testing | Diagnostic cost | Costs in functional testing group ¼ (0.94 × E exercise ECG) + (0.29 × E SPECT) + (0.06 × E CCTA) + (0.07 × E Stress echo) + (0.11 × E Cath) Costs in CT group ¼ (1 × E CAC-score) + (0.46 × E CCTA) + (0.07 × E SPECT) + (0.05 × E exercise ECG) + (0.01 × E Stress echo) + (0.12 × E Cath) | CT vs. functional testing<br>€369 vs. €440 --> cumulative diagnostic costs | Lubbers M et al[6] |
| n/a | 2019 | CT vs. SPECT | 1 year | Cost-effectiveness analysis | CT vs. SPECT (USD 4514 v 5208) --> per patient<br>QALYs<br>(0.938 vs. 0.955, P-value = 0.039) | Lee SP [7] |
| n/a | 2012 | CT vs. myocardial perfusion single-photon emission CT (MPS) | Diagnostic cost | | CT vs. MPS ($781.08 (IQR, $367.80-$4349.48] v $1214.58 [IQR, $978.02-$1569.40] | Min JK et al[8] |
| n/a | 2008 | CT vs. standard of care (SOC) | | Microsimulation model (Analyses for 55-year-old men and women with "low-risk chest pain) | Using CT coronary angiography to triage men with acute chest pain increased emergency department and hospital costs by $110 and raised total health care expenditures by$200. In women, the technology was cost-saving, and emergency department and hospital costs decreased by $410; total health care expenditures decreased by $380. | Ladapo J.A et al[9] |
| n/a | 2012 | CT vs. standard of care (SOC) | Short-term (12 month) | Cost-minimization model | Modeled mean patient costs for CCT-based evaluation were $750 (24%) lower than the SOC ($2,384 and $3,134, respectively) | Branch K. R. et al[10] |

## References


1. Chang HJ, Lin FY, Gebow D, et al. Selective Referral Using CCTA Versus Direct Referral for Individuals Referred to Invasive Coronary Angiography for Suspected CAD: A Randomized, Controlled, Open-Label Trial. *JACC Cardiovasc Imaging* 2019;12(7 Pt 2):1303-12. doi: 10.1016/j.jcmg.2018.09.018 [published Online First: 2018/12/17]
2. Rudziński PN, Kruk M, Kępka C, et al. Assessing the value of coronary artery computed tomography as the first-line anatomical test for stable patients with indications for invasive angiography due to suspected coronary artery disease. Initial cost analysis in the CAT-CAD randomized trial. *J Cardiovasc Comput Tomogr* 2020;14(1):75-79. doi: 10.1016/j.jcct.2019.07.008 [published Online First: 20190725]
3. Thokala P, Goodacre S, Oatey K, et al. Cost-effectiveness of rapid assessment of potential ischaemic heart disease with CT coronary angiography. *Heart* 2023;109(6):464-69. doi: 10.1136/heartjnl-2022-321211 [published Online First: 20230223]
4. Goldstein JA, Gallagher MJ, O'Neill WW, et al. A randomized controlled trial of multi-slice coronary computed tomography for evaluation of acute chest pain. *J Am Coll Cardiol* 2007;49(8):863-71. doi: 10.1016/j.jacc.2006.08.064 [published Online First: 20070212]
5. Mark DB, Federspiel JJ, Cowper PA, et al. Economic Outcomes With Anatomical Versus Functional Diagnostic Testing for Coronary Artery Disease. *Ann Intern Med* 2016;165(2):94-102. doi: 10.7326/m15-2639 [published Online First: 2016/05/24]
6. Lubbers M, Dedic A, Coenen A, et al. Calcium imaging and selective computed tomography angiography in comparison to functional testing for suspected coronary artery disease: the multicentre, randomized CRESCENT trial. *Eur Heart J* 2016;37(15):1232-43. doi: 10.1093/eurheartj/ehv700 [published Online First: 20160107]
7. Lee SP, Seo JK, Hwang IC, et al. Coronary computed tomography angiography vs. myocardial single photon emission computed tomography in patients with intermediate risk chest pain: a randomized clinical trial for cost-effectiveness comparison based on real-world cost. *Eur Heart J Cardiovasc Imaging* 2019;20(4):417-25. doi: 10.1093/ehjci/jey099
8. Min JK, Koduru S, Dunning AM, et al. Coronary CT angiography versus myocardial perfusion imaging for near-term quality of life, cost and radiation exposure: a prospective multicenter randomized pilot trial. *J Cardiovasc Comput Tomogr* 2012;6(4):274-83. doi: 10.1016/j.jcct.2012.06.002 [published Online First: 20120611]
9. Ladapo JA, Hoffmann U, Bamberg F, et al. Cost-effectiveness of coronary MDCT in the triage of patients with acute chest pain. *AJR Am J Roentgenol* 2008;191(2):455-63. doi: 10.2214/ajr.07.3611
10. Branch KR, Bresnahan BW, Veenstra DL, et al. Economic outcome of cardiac CT-based evaluation and standard of care for suspected acute coronary syndrome in the emergency department: a decision analytic model. *Acad Radiol* 2012;19(3):265-73. doi: 10.1016/j.acra.2011.10.029 [published Online First: 20111230]
